# Cohort profile: the West-China hospital alliance longitudinal epidemiology wellness (WHALE) study

**DOI:** 10.1007/s10654-025-01290-1

**Published:** 2025-08-23

**Authors:** Yifei Lin, Yong Yang, Zhuyue Li, Liang Du, Rui Shi, Qingke Shi, Xueru Xu, Geng Yin, Fan Zhang, Wenxia Huang, Yan Huang, Ga Liao, Qilin Liu, Weimin Li, Huan Song, Jin Huang

**Affiliations:** 1 Health Management Center, General Practice Medical Center, Innovation Institute for Integration of Medicine and Engineering, West China Hospital, Sichuan University, Chengdu, Sichuan China; 2 General Practice Medical Center, and Chinese Evidence-Based Medicine Center, West China Hospital, Sichuan University, Chengdu, Sichuan China; 3 Engineering Research Center of Medical Information Technology, Ministry of Education, West China Hospital, Sichuan University, Chengdu, Sichuan China; 4 Department of Orthopedics, West China Hospital, Sichuan University, Chengdu, Sichuan China; 5 Department of General Practice, General Practice Medical Center, West China Hospital, Sichuan University, Chengdu, Sichuan China; 6 State Key Laboratory of Oral Diseases, National Clinical Research Center for Oral Diseases, West China Hospital of Stomatology, Sichuan University, Chengdu, Sichuan China; 7 Department of Pulmonary and Critical Care Medicine, West China Hospital, State Key Laboratory of Respiratory Health and Multimorbidity, West China Hospital, Sichuan University, Chengdu, Sichuan China; 8 Institute of Respiratory Health, Frontiers Science Center for Disease-related Molecular Network, West China Hospital, Sichuan University, Chengdu, Sichuan China; 9 West China Biomedical Big Data Center, West China Hospital, Sichuan University, Chengdu, Sichuan China; 10 Present Address: Department of Urology, Lab of Health Data Science, West China hospital, Sichuan University, Chengdu, Sichuan, China

**Keywords:** Longitudinal cohort study, Health check-up, Health trajectory, Multi-omics, Proactive health, Big data

## Abstract

The West-China Hospital Alliance Longitudinal Epidemiology Wellness (WHALE) Study establishes a robust, multidimensional database to provide comprehensive insights into health-to-disease transitions, advancing proactive healthcare and enhancing understanding of the interplay among genetic, behavioral, and environmental factors in disease. The WHALE Study includes a database and a cohort. The WHALE Database, established in 2010, integrates health check-up data from 11 hospitals, covering sociodemographic, lifestyle, medical history, and clinical data. The WHALE Health Trajectory Cohort, launched in November 2024, recruits adults with at least three health check-ups, featuring biennial active follow-ups and passive linkage with regional healthcare databases. As of January 2025, the WHALE Database includes over 3.4 million health records from 1,526,686 participants, with a mean age of 40.3 years and a balanced gender distribution. Notably, 23.88% of participants had at least three health check-ups, and 3.31% had more than ten, highlighting a significant proportion with repeated measurements. The study provides key insights into health trajectories by examining the associations of biomarker data and their trajectory patterns with aging, pre-disease conditions, and disease diagnoses. The strengths of the WHALE Study include its large sample size, longitudinal design, diverse representation, comprehensive data, and robust quality control. Limitations include potential selection bias, data variability across centers, and reliance on self-reported data for some variables.

**Supplementary Information:**

The online version contains supplementary material available at 10.1007/s10654-025-01290-1.

## Introduction

In response to the persistent challenges and the significant global burden posed by unfavorable behaviors, diseases, and injuries, healthcare is shifting from a reactive to a proactive paradigm [1, 2]. This transition emphasizes comprehensive and early preventive strategies, encompassing timely screening, accurate diagnosis, and effective treatment. By identifying preclinical conditions and leveraging dynamic health metrics, the healthcare system is increasingly able to implement early interventions, addressing not only the impacts of pandemics and chronic diseases but also conditions like cancer at their earliest stages, before progression occurs.

In this context, the study of health metric trajectories, encompassing both clinical measurements and biomarkers, has gained accumulating attention in population-based research [3–5]. An in-depth understanding of these health-to-disease trajectories can inform the development of optimized models to promote individual wellness across the lifespan and throughout the entire health cycle. From a population perspective, this proactive paradigm provides widespread health benefits while remaining the most cost-effective method to enhance public health and improve healthcare systems [6]. For instance, some studies, primarily utilizing data from healthcare registries, have modeled health trajectories to uncover patterns in health metrics preceding disease onset and to investigate associations with potential risk factors such as genetics, lifestyle, and environmental influences [7–9]. However, these registries typically collect only selective measures based on reported symptoms or clinical indications during primary care visits or hospitalizations, resulting in limited data coverage. Studies that leveraged repeated health check-up data, which captures comprehensive and multidimensional phenotypes across various time points, remain scarce [10, 11].

### General and specific objectives

In China, health check-up services are mainly provided by hospitals, which are also the primary provider of disease-oriented medical services [12]. Organization-based group screenings and mandatory exams for employment, school, and military service account for 70−80% of health check-ups [13]. The hospital-centered health check-ups present a unique advantage, as data collected for health assessments and disease treatment are in the same healthcare setting. This abundance of data offers substantial potential for building robust, multidimensional databases and cohorts that track health trajectories across the population. Nevertheless, pooling and standardizing this data across multiple hospitals poses a significant challenge, especially given the diversity in laboratory instruments, imaging technologies, and data management systems employed at various healthcare facilities.

The West-China Hospital Alliance [14–16], a consortium of 11 hospitals sharing standardized staff qualifications, equipment, and data management protocols, offers a unique chance to address the needs for obtaining continuous, high-quality health trajectory data through the initiation of the West-China Hospital Alliance Longitudinal Epidemiology Wellness (WHALE) study. The WHALE database aggregates data from alliance hospitals with a pre-established quality check process, creating a reliable and detailed repository that includes sociodemographic profiles, lifestyle factors, medical and clinical intervention histories, laboratory biomarkers, and imaging data. This extensive dataset al.lows for the exploration of health outcomes and risk factors within a unified framework.

Building on the WHALE database, the WHALE Health Trajectory Cohort was established on November 1, 2024, to systematically analyze how health metrics and biomarkers, such as genetic markers, lifestyle, diet, and psychological factors, along with their dynamic changes over time, impact the onset, progression, and prognosis of a wide array of health conditions. Unlike the WHALE database, which primarily consolidates data generated from routine healthcare interactions, this cohort incorporates an active follow-up plan to continuously engage individuals who have not returned for regular health check-ups. Additionally, we enriched the phenotype collections and linked to regional health-related administrative databases to capture long-term health outcomes comprehensively. The WHALE Health Trajectory Cohort aims to offer insights into precise preventive healthcare strategies (e.g., personalized health promotion plans) and enhance understanding of the complex interactions between genetic, behavioral, and environmental factors in chronic disease development.

## Methods

### Study design and population

The WHALE Study includes two main components: the WHALE Database and the WHALE Health Trajectory Cohort. The WHALE Database, established in 2010, integrates health check-up data from 11 hospitals in Sichuan Province. The WHALE Health Trajectory Cohort, launched in November 2024, recruits adults with at least three health check-ups, focusing on those living in Chengdu and using 6 specific centers for data collection. Inclusion criteria are adults aged ≥ 18 years with multiple health check-ups. Exclusion criteria include incomplete data or lack of consent for follow-up.

The rationale for including only participants with at least three health check-ups in the WHALE Health Trajectory Cohort is primarily to ensure robust longitudinal analyses. Specifically, having at least three repeated assessments provides essential data points needed to reliably characterize individual-level health trajectories over time. This criterion significantly enhances our ability to detect subtle yet clinically meaningful changes in health metrics, facilitating earlier identification of disease markers and pre-disease conditions. Moreover, multiple repeated measures allow us to better distinguish true longitudinal trends from random fluctuations or measurement errors, improving analytical precision and interpretability. Additionally, participants with multiple check-ups tend to demonstrate better adherence to health monitoring programs, reflecting greater health awareness and motivation. Such individuals typically exhibit more stable residential patterns and occupational conditions, further enhancing data completeness, consistency, and long-term follow-up reliability.

### Informed consent

was obtained from all participants in accordance with the Declaration of Helsinki. Beginning in 2015, a standardized broad consent form has been implemented across WHALE study sites. Participants were informed about the use of their clinical and questionnaire data, biospecimen collection, long-term storage, and future research use. Broad consent was obtained at each health check-up visit, including the baseline visit, with re-consenting during subsequent follow-ups. Participants were also informed of their right to withdraw at any time. Blood samples have been collected since 2015, and urine samples since 2025, under the same broad consent framework. The study protocol was approved by the ethics committee of West China Hospital, Sichuan University (approval number 2015-292; 2022-462; 2024-2012).

### Data collection

#### Baseline information

**Fig. 1:**
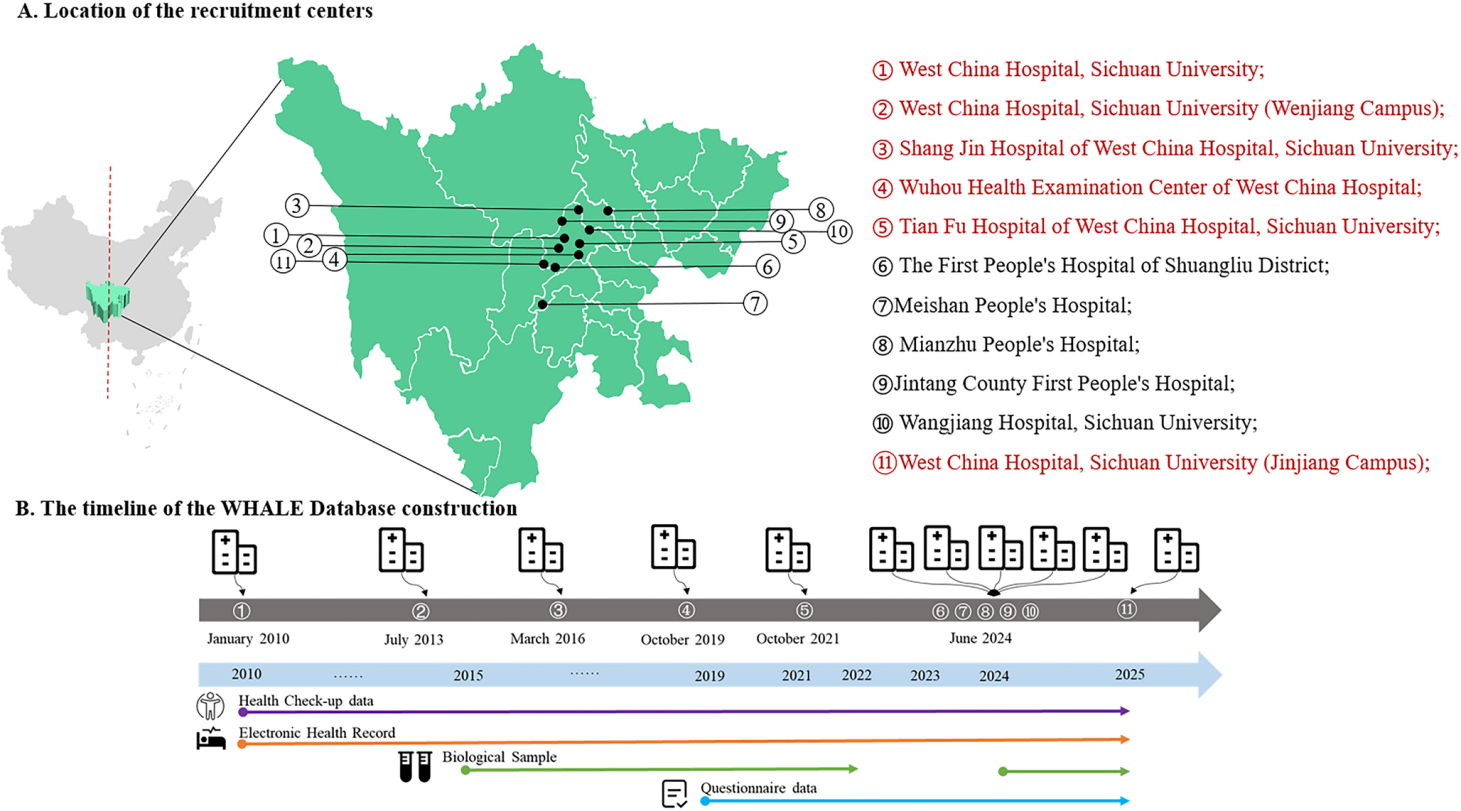
Location of involved West-China Hospital Alliance hospitals and timeline of WHALE Study-Database construction. Institutions marked in red represent the participating hospitals in the Health Trajectory Cohort study

The WHALE Database includes individuals who received health check-ups at so far 11 hospitals of West-China Hospital Alliance members in Sichuan Province—a region connecting western and eastern China (Fig. 1). It was initially established at West China Hospital, Sichuan University on January 1 st 2010, and then gradually started integrating data from another nine alliance members (joined between 2013 and 2025, see Supplementary Table 1 for details). To ensure data comparability, we first evaluated the consistency of used laboratory instruments, imaging technologies, and data management systems in the alliance hospitals with the West China Hospital, Sichuan University, to decide the priority of data integration. Also, we evaluated their health check-up database structures of alliance hospitals and assessed the network interoperability to confirm data traceability. After data collection at each participating hospital, all records were securely transmitted to the Department of Information Technology at West China Hospital, Sichuan University, via a dedicated network line providing secure, stable, and high-speed data transfer. Before being entered into the WHALE database, all records underwent rigorous cleaning and quality control on the Big Data Platform [17] developed by West China Hospital, Sichuan University (see Supplementary Fig. 1), ensuring high reliability and consistency. Beginning in 2015, participants were asked to donate a blood sample (residual from routine laboratory tests), and a screening questionnaire (60 items for males and 58 items for females) was introduced in 2019 to collect detailed information on demographics, lifestyle (smoking, alcohol drinking, dietary preference, physical activity, and sedentary behavior), sleep quality, and medical history. Additionally, an option for psychological evaluation (measuring symptoms of anxiety and depression) became available in 2019 for health check-up individuals.

The WHALE Study released its first dataset in March 2022, encompassing 478,898 individuals from West China Hospital, Sichuan University [15]. The second dataset was released in February 2023, including 685,163 participants. As of January 15th 2025, data from 11 hospitals have been consolidated, comprising 3,466,269 records from 1,526,686 unique individuals including 8,00,064 records from the main hospital. Notably, 23.88% of participants underwent health check-ups more than three times, 12.03% had more than five times, and 3.31% more than 10 times, demonstrating a substantial proportion with repeated measurements (Supplementary Table 2). Additionally, linked data from clinical medical records were available for over 510,000 participants, enabling tracking of disease outcomes.

**Fig. 2:**
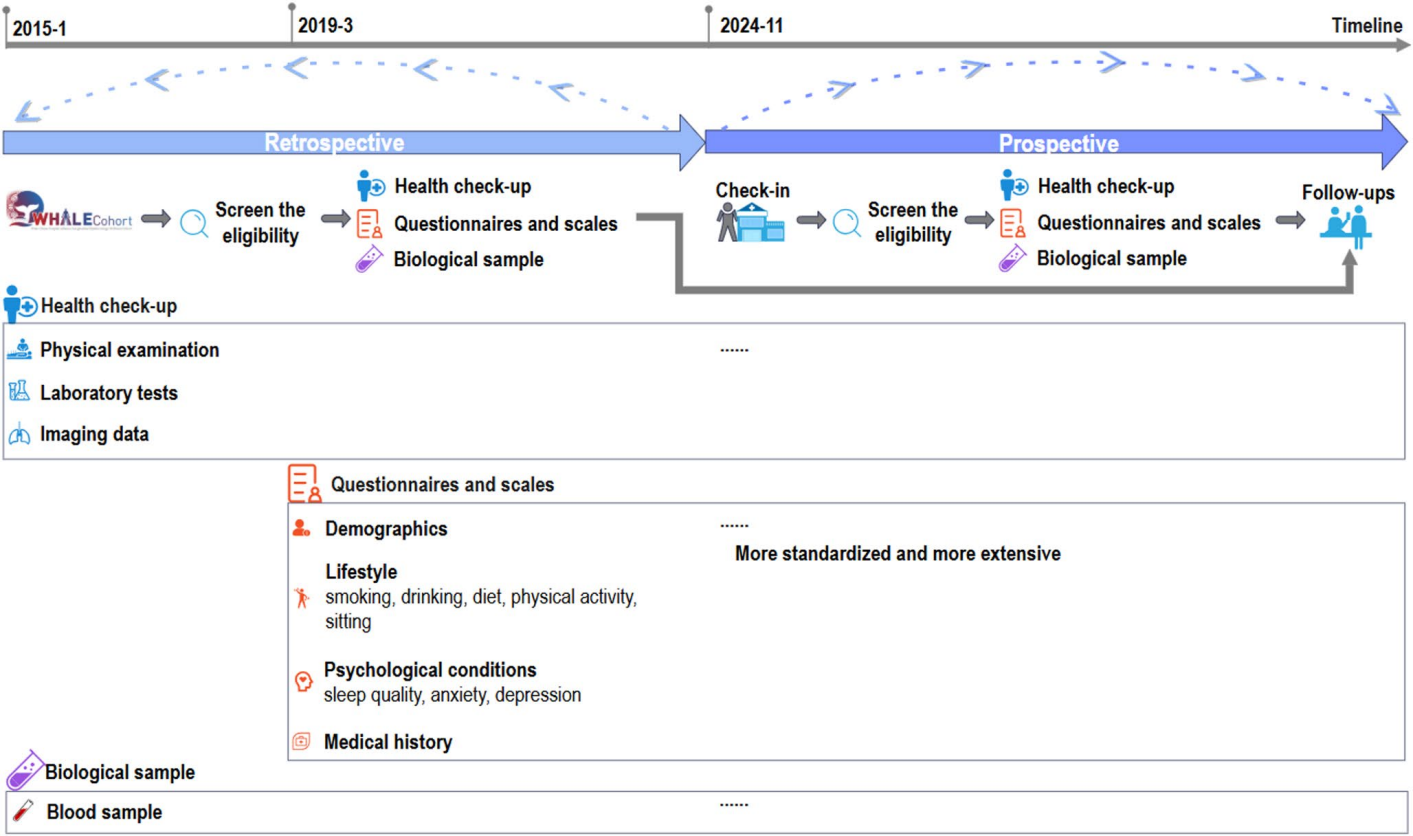
The ambispective cohort design of the WHALE Study- Health Trajectory Cohort

The WHALE Health Trajectory Cohort is an ongoing ambispective cohort study established in November 1 st 2024 (Fig. 2), which historically (for participants included between January 1 st 2015 and October 31 st 2024) and prospectively (November 1 st 2024 onwards) recruited adults (≥ 18 years) having three health check-up records in the WHALE Database (i.e., the baseline date was the date of their third check-up). Align with the accessibility of obtaining health outcome data from regional healthcare registers, we limited our participants as those living in Chengdu (lived more than 5 years) and conducted the third health check-up at six centers of West-China Hospital Alliance members located in Chengdu (Fig. 1, hospitals ①-⑤, ⑪). Besides abovementioned health metrics in the WHALE Database, we revised and extended the original screening questionnaires, with more detailed information collected for diet, physical activity, and psychological symptoms. The questionnaire was sent to participants in the early morning of their health check-up day in the prospective cohort, together with a request for blood and urine donation. The check-in/check-out nurse was responsible for confirming the willingness of participation (for the bio-sample collection arrangement), helping with any inquires and checking the completeness of questionnaires. The historical cohort included 273,628 adults, with 105,402 having denated blood samples. The ongoing prospective cohort recruited 2,880 adults until January 15th, 2025.

### Follow-ups

**Fig. 3:**
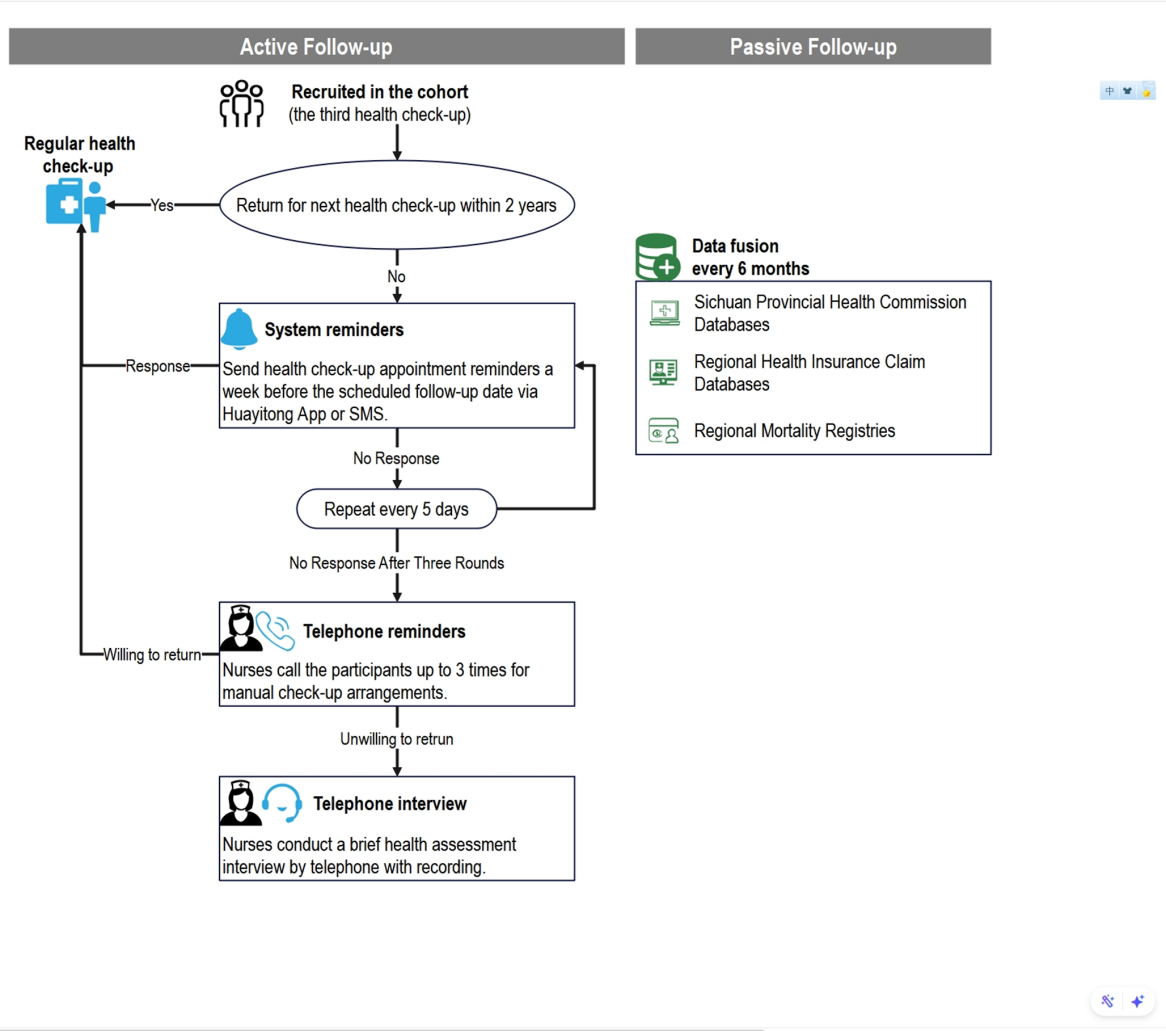
The follow-up schedule of the WHALE Study- Health Trajectory Cohort

Participants recruited through both historical and prospective approaches in the WHALE Health Trajectory Cohort are enrolled in a comprehensive follow-up protocol that combines active and passive data collection (Fig. 3). Active follow-up is conducted biennially, designed to ensure continuous monitoring of participants’ health trajectories. In brief, for those who do not return within two years of their last health check-up, an automated reminder system (a customized Cohort Data Collection and Management System integrated within the Health Check-up systems) initiates a sequence of notifications, beginning one week before the scheduled follow-up date. A reminder message, along with a link to schedule a new health check-up, is sent via text message or the hospital’s patient registration and appointment system, the Huayitong App. If no response is received, additional reminders dispatched at five-day intervals. If participants still do not respond, follow-up nurses make up to three phone calls to arrange a check-up manually or, alternatively, to conduct a brief health assessment interview, recording health status and reasons for non-return (Fig. 3). Non-responders who remain unreachable after three attempts, or who explicitly decline follow-up, are documented, and this active follow-up cycle for them is closed. Next follow-up cycle for each participant continued even if a prior follow-up cycle is missed or non-responsive. The follow-up only terminates upon death or a withdrawal of informed consent.

The WHALE Study employs robust systems for passive follow-up to ensure comprehensive tracking of long-term health outcomes across the cohort. These systems enable reliable linkage of health outcomes, including for participants lost to active follow-up. Specifically, mortality data are drawn from the national death registration system managed by the China CDC, which aggregates cause-of-death data from 31 mortality surveillance points across Sichuan [18]. Disease diagnoses are obtained from the Sichuan Health Statistics Reporting System, which compiles first-page inpatient records from all regional hospitals [19]. Together, these linkages ensure robust passive follow-up every six months.

### Phenotypic measurement data

**Fig. 4:**
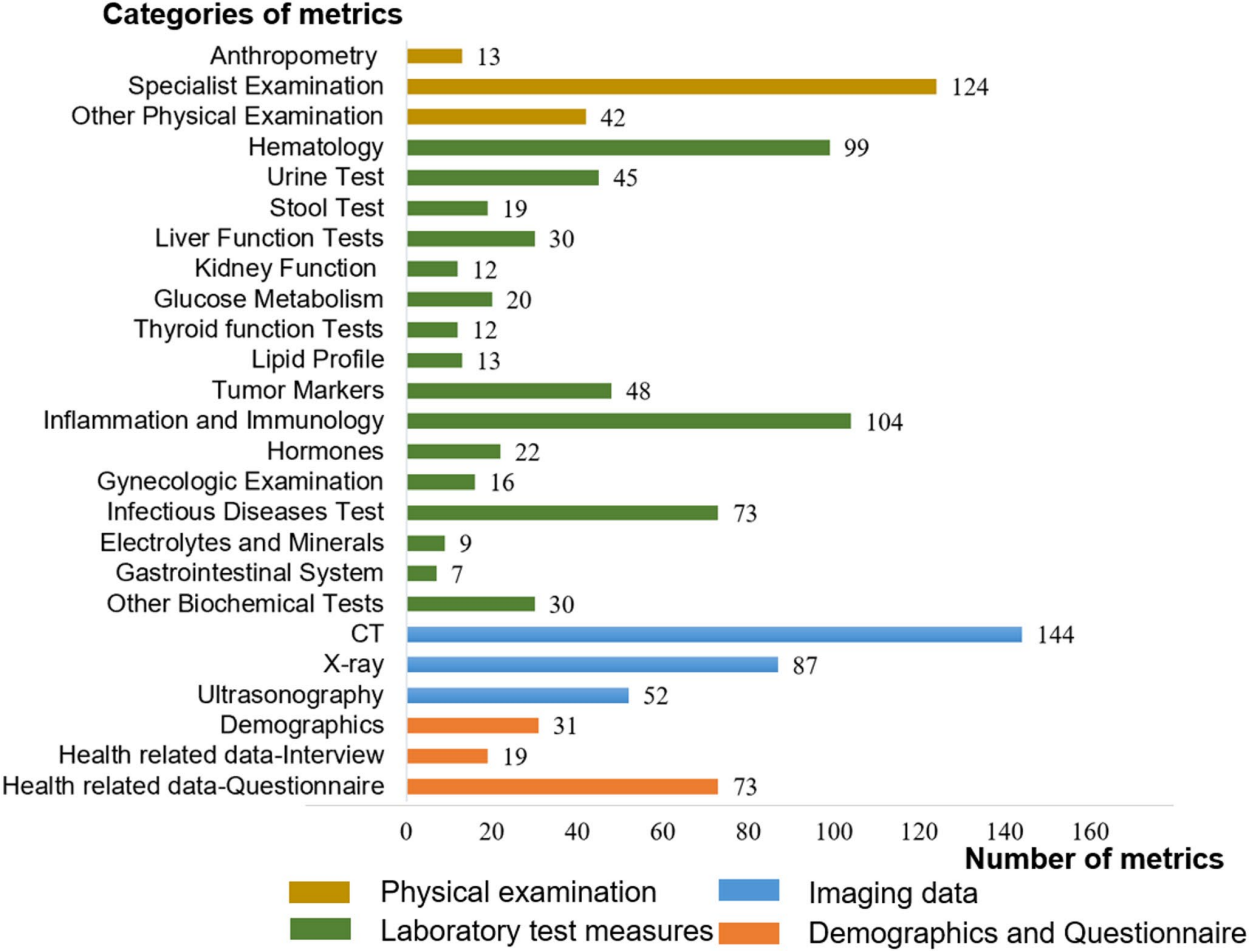
The number of metrics in selective categories from the WHALE Study-Database

The WHALE Database comprises a total of 1083 health check-up metrics across 22 major categories, encompassing data from physical examinations, laboratory tests, imaging studies, and questionnaires. However, the specific items recorded for each participant vary based on the examination packages selected by employer-organized annual check-ups or the personalized choices of those undergoing individual health assessments. Figure 4 and Supplementary Table 3 lists a selection of representative health metrics from the WHALE Database. Detailed equipment information used in the health examination, laboratory tests, and imaging test can be found in Supplementary Table 4.

#### Physical examination

Anthropometric measurements, including body weight and height, were precisely taken using the ultrasonic auto-anthropometer SG-1001SC (Chioy, China), with participants dressed in light indoor clothing and barefoot. Waist circumference was measured at midpoint between the lower rib margin and the iliac crest during minimal respiration, while hip circumference was taken at the widest point around the pelvis, aligned with the greater trochanter of the femur. Blood pressure, including systolic and diastolic values, was measured on the right arm of the seated subjects after a 5-minute rest, using the tunnel-style ABP-1000 (F-version) blood pressure monitor (Chioy, China), to ensure accurate and consistent readings.

Specialist assessments covered internal medicine, surgery, dentistry, and otorhinolaryngology. During internal exams, patients lie supine with knees slightly bent for heart and lung auscultation and abdominal palpation to check for tenderness, rebound pain, and masses. Surgical phycial examination, including visual inspection and palpation, was conducted to assess on the skin, lymph nodes, spine, breasts, and urogenital areas. Dentists and otorhinolaryngologists conducted general assessments of oral and upper respiratory health, while ophthalmologists performed slit-lamp examinations to assess the eyelids, conjunctiva, cornea, lens, and other ocular tissues. Ophthalmic examination images are all properly stored in the Picture Archiving and Communication System database owned by department of information technology of West China Hospital.

Other important Physical examinations include bone density measurements (MetriScan, Miles Medical LLC, China), body composition analysis (InBody570, InBody, Korea), liver assessments (FibroScan 502 Touch, ECHOSENS, France) for fatty liver and fibrosis evaluation, pulmonary function tests (MasterScreen SeS, Jaeger, Germany). A 12-lead electrocardiogram (iMAC120, ZONCARE, China) diagnoses cardiac arrhythmias, capturing data such as heart rate, P wave, and PR interval. All tests are performed with specialized equipment following standardized procedures. (Supplementary Table 4).

#### Laboratory test measures

Following a 10–12 h overnight fast, peripheral blood samples were collected in the early morning by skilled nursing staff at the Health Management Center of West China Hospital. Urine and stool samples were also obtained after fasting overnight. All laboratory tests were conducted in the hospital’s clinical laboratory in adherence with established protocols.

Beyond routine tests for hematology, urine, stool analysis, as well as liver, kidney, thyroid function and glucose and lipid metabolism, the WHALE health check-up protocol includes an wider range of specialized assays. Using standard instruments and established protocols, we measure a range of biomarkers with varying coverage among participants, including tumor markers (e.g., carcinoembryonic antigen, alpha-fetoprotein, prostate-specific antigen, carbohydrate antigen 19 − 9, and carbohydrate antigen 15 − 3), inflammation and immunology markers (e.g., C-reactive protein, erythrocyte sedimentation rate, anti-nuclear antibody, rheumatoid factor, and immunoglobulin M), and sex hormones (e.g., testosterone, estradiol, progesterone, follicle stimulating hormone, and luteinizing hormone) (Supplementary Table 4).

#### Imaging data

Participants underwent a comprehensive set of imaging examinations, including ultrasonography, X-rays, chest CT scans, and electrocardiogram. Ultrasonography (EPIQ5, PHILLIPS, USA) was used to examine the morphology, size, and position of internal organs and tissues, as well as to detect abnormal masses such as stones and tumors. Key areas evaluated include the liver, gallbladder, kidneys, spleen, thyroid, and cervical lymph nodes. For optimal imaging, a gel layer was applied to the abdominal area, and the clinician guided the transducer across the skin to capture detailed images. Chest X-rays (uDR780i, United-Imaging, China) were performed primarily to assess cardiopulmonary health, monitoring for infections, tuberculosis, pulmonary nodules, and heart morphology. During the scan, participants stood facing the receptor plate, with their back to the X-ray source, hands behind their back, shoulders relaxed, and chin resting on the plate. Chest CT scans (SOMATOM Definition AS 128 scanner, Siemens, Germany) focused on detecting abnormalities in the lungs, pleura, and mediastinum. Participants could also opt for additional scans of other areas, such as the extremities or abdomen, as required.

#### Questionnaire data

The health check-up screening questionnaire employed in 2019 included 60 questions for males and 58 questions for females, covering demographics information (11 items), lifestyle factors (e.g., smoking, drinking, diet, physical activity, and sedentary behavior, 21 items), assessment of sleep quality (1 item), medical and family history of chronic diseases (i.e., hypertension, cardiovascular diseases, diabetes, hyperlipemia, cancer) (9 items), and medication use history (frequency and duration of antihypertensive drugs, antidiabetic drugs, and antihyperlipidemic drugs, 3 items). Besides, selected participants were evaluated for anxiety and depression symptoms (40 item), as well as further evaluation of sleep quality (19 items). We listed detailed questions/scales and responses in Supplementary Table 5.

In the prospective phase of the WHALE Health Trajectory Cohort, the health check-up questionnaire was expanded to include additional items on diet habits (7 new items) and updated scales for psychological assessments, using the 9-item Patient Health Questionnaire-9 [20] for depressive symptoms and 7-item Generalized Anxiety Disorder Scale [21] for anxious symptoms). Likewise, physical activity assessment was improved by replacing self-designed questions with the Chinese version of the International Physical Activity Questionnaire short form [22].

During the biennial active follow-up, if participants decline to return for a regular health check-up, follow-up nurses conduct a brief 25-item health assessment through telephone interviews. This questionnaire mainly captures information on common diseases and medications, inquiring about any new diseases and medication used over the 2-year interval without an in-person check-up.

### Blood sample management

A total of 177,467 plasma samples and 185,759 buffy coat samples were extracted from the remaining blood collected during health check-ups between 2015 and 2022, due to storage limitations of the West China Biobank. Biospecimen collection followed standardized protocols to ensure biomarker stability and reliability for downstream analyses. After initial laboratory tests conducted by the Department of Laboratory Medicine, the remaining blood samples were promptly transported on ice to the sample bank laboratory by designated personnel within 15–20 min to maintain stability. Samples were then centrifuged at 4 °C and 1600 g for 15 min. Technicians inspected the plasma supernatant for any unusual conditions, such as hemolysis or lipemia. Using the fully automated TECAN Freedom EVO200 system, plasma and buffy coat were separated from EDTA, heparin, or sodium citrate anticoagulated blood. Barcodes on tubes and cryovials were scanned, and blood component interfaces were measured automatically. The system precisely aliquoted plasma and buffy coat into two portions, with buffy coat tubes containing ~ 300 µL and plasma volumes ranging from 13 µL to 1179 µL (see Supplementary Fig. 2). Cryovial caps were sealed, and samples were stored at −80 °C before being transferred on dry ice to the central repository at West China Biobank [23] for long-term preservation.

Between April 26th 2021, and November 25th 2022, using previous blood samples from 7,084 hospital employees, DNA was successfully extracted and whole genome sequencing was performed by Precision Medicine Center of West China Hospital, Sichuan University. The sequencing data were derived from the NovaSeq 6000 platform (Illumina, USA), with a target coverage depth of 30×.

With the expansion of our biobank storage capacity, we aim to continue collecting and storing new blood and urine samples following a standardized protocol started from 2025. For blood collection, one 6 mL fasting venous blood sample (EDTA) and one 5 mL venous blood sample (yellow-top) will be drawn, processed within two hours, centrifuged, and stored at −80 °C, with monthly transfers to the West China Biobank. Similarly, a 10 mL urine sample will be collected, processed, centrifuged, and stored under the same conditions, ensuring systematic long-term preservation.

### Quality control

The WHALE Study employs a robust, two-staged quality control framework to ensure data integrity across health check-up and diagnostic data sources (Supplementary Fig. 1). A centralized data dictionary was developed to standardize data elements across the 11 West China Hospital Alliance sites. These layered processes enhance the reliability, internal consistency, and cross-site comparability of data for both cross-sectional and longitudinal analyses.

**Stage 1 – Local validation** Health check-up records collected by trained clinicians were subject to real-time automated checks through the West-China Hospital Alliance hospitals system. Implausible or missing values were flagged for immediate review and correction by site personnel.

**Stage 2 – Central audit** After secure transmission to the West China Hospital Big Data Platform, diagnostic data (including ICD-10 coded entries from alliance hospitals and the Sichuan Health Statistics Reporting System) underwent centralized harmonization and automated validation. A dedicated governance team of five full-time staff, evaluated seven data quality dimensions—completeness, validity, uniqueness, consistency, precision, logical coherence, and usability—based on the Plan-Do-Check-Act (PDCA) framework [24] (Supplementary Fig. 3). A 10% random sample was manually audited for accuracy and internal consistency. Free-text diagnoses were standardized to ICD-10 codes to ensure uniform classification.

## Results

### Basic characteristics of the participants

Table 1 provides an overview of the basic characteristics of participants in the WHALE Study. In brief, for all participants included in the database, the average age at the first health check-up was 40.30 years, with a balanced gender distribution (46.8% male). The majority of participants were Han ethnicity (85.6%); however, due to the large sample size of this database, there was also a significant number of ethnic minorities (2.5%). Most participants were married (64.2%) and had a relatively high educational level (28.0% having a bachelor’s degree). With much smaller proportions of missing data, the baseline characteristics of individuals in the WHALE Health Trajectory Cohort were largely consistent, with an average recruitment age of 39.75 years, a comparable gender balance (45.3% male), and similar patterns in ethnicity, marital status, educational attainment, Body Mass Index and smoking or drinking status. To assess potential bias from the inclusion criterion, we compared baseline characteristics between the full WHALE Database and the Health Trajectory Cohort (≥ 3 check-ups). While differences were observed, these were in line with anticipated patterns of follow-up engagement and were taken into account in the interpretation of findings (Table 1).

**Table 1:** Basic characteristics of participants in the WHALE database and the WHALE health trajectory cohort

Characteristics	The WHALE Database (January 1 st 2010–January 15th 2025)				The WHALE Health Trajectory Cohort (January 1 st 2015–January 15th 2025)
	Overall (*N* = 1,526,686)	Health check-up times			Overall (*N* = 276,508)
		1 to 2 times (*N* = 1,162,043)	3 to 9 times (*N* = 314,034)	≥ 10 times (*N* = 50,609)	
Health check-up types, No.(%)					
Organization-based group check-up	1,033,682(67.7)	703,653(60.6)	279,997(89.2)	50,032(98.9)	251,589(91.0)
Individual check-up	493,004(32.3)	458,390(39.4)	34,037(10.8)	577(1.1)	24,919(9.0)
Age at first health check-up, years, Mean (SD)	40.30(14.60)	40.11(14.95)	40.74(13.43)	41.86(13.15)	39.75(12.54)
Sex, No. (%)					
Male	807,125(52.9)	606,441(52.2)	174,499(55.6)	26,185(51.7)	151,303(54.7)
Female	715,048(46.8)	551,090(47.4)	139,534(44.4)	24,424(48.3)	125,205(45.3)
Unknown	4513(0.3)	4512(0.4)	1(0.0)	0(0.0)	0(0.0)
Ethnicity, No. (%)					
Han	1,306,727(85.6)	983,748(84.7)	276,703(88.1)	46,276(91.4)	270,052(97.7)
Non-Han	37,780 (2.5)	31,070 (2.6)	6443 (2.1)	56(0.6)	4886 (2.3)
Unknown	182,179(11.9)	147,225(12.7)	30,888(9.8)	4,066(8.0)	0(0.0)
Marriage status, No. (%)					
Single	265,332(17.4)	203,518(17.5)	53,616(17.1)	8,198(16.2)	49,590(17.9)
Married	980,612(64.2)	713,375(61.4)	229,831(73.2)	37,406(73.9)	219,335(79.3)
Divorced	2,225(0.1)	1,604(0.1)	585(0.2)	36(0.1)	568(0.2)
Widowed	175(0.0)	123(0.0)	51(0.0)	1(0.0)	24(0.0)
Unknown	278,342(18.2)	243,423(20.9)	29,951(9.5)	4,968(9.8)	6,991(2.5)
Education level, No. (%)					
Primary school	13,689(0.9)	12,205(1.1)	1,375(0.4)	109(0.2)	1,371(0.5)
Middle school	36,014(2.4)	28,595(2.5)	6,617(2.1)	802(1.6)	6,955(2.5)
High school	60,460(4.0)	40,126(3.5)	16,553(5.3)	3,781(7.5)	19,503(7.1)
Bachelor degree	427,220(28.0)	245,937(21.2)	149,999(47.8)	31,284(61.8)	177,797(64.3)
Postgraduate degree or above	92,231(6.0)	49,056(4.2)	35,916(11.4)	7,259(14.3)	42,659(15.4)
Unknown	897,072(58.8)	786,124(67.7)	103,574(33.0)	7,374(14.6)	28,223(10.2)
Occupation, No.(%)					
Unemployed	31(0.0)	27(0.0)	3(0.0)	1(0.0)	0(0.0)
Agricultural or industry workers	20,489(1.3)	17,995(1.5)	2,274(0.7)	220(0.4)	2,325(0.8)
Enterprise staffs	257(0.0)	59(0.0)	100(0.0)	98(0.2)	196(0.1)
Professional and technical personnel	9,731(0.6)	2,768(0.2)	4,508(1.4)	2,455(4.9)	1,326(0.5)
Administrative and managerial staffs	76,837(5.0)	38,904(3.3)	31,259(10.0)	6,674(13.2)	36,975(13.4)
Others ^b^	586,536(38.4)	383,195(33.0)	171,170(54.5)	32,171(63.6)	196,850(71.2)
Retired	62,554(4.1)	36,069(3.1)	18,690(6.0)	7,795(15.4)	21,666(7.8)
Unknown	770,251(50.5)	683,026(58.8)	86,030(27.4)	1,195(2.4)	17,170(6.2)
Body Mass Index, BMI, No. (%)					
< 18.5 kg/m^2^	86,906(5.7)	64,924(5.6)	18,924(6.0)	3,058(6.0)	17,516(6.3)
18.5–23.9 kg/m^2^	66,9035(43.8)	492,079(42.3)	150,877(48.0)	26,079(51.5)	143,225(51.8)
24.0–27.9 kg/m^2^	381,786(25.0)	283,484(24.4)	84,366(26.9)	13,936(27.5)	78,999(28.6)
≥ 28.0 kg/m^2^	114,172(7.5)	88,439(7.6)	22,616(7.2)	3117(6.2)	20,743(7.5)
Unknown	274,787(18.0)	233,117(20.1)	37,251(11.9)	4419(8.7)	16,025(5.8)
Smoking status, No.(%)					
Never smoke	707,565(46.3)	505,648(43.5)	170,799(54.4)	31,118(61.5)	174,813(63.2)
Current smokers	262,376(17.2)	176,364(15.2)	72,509(23.1)	13,503(26.7)	70,619(25.5)
Past smokers	29,116(1.9)	19,742(1.7)	8067(2.6)	1307(2.6)	7,736(2.8)
Unknown	527,629(34.6)	460,289(39.6)	62,659(20.0)	4681(9.2)	23,340(8.4)
Drinking habits, No.(%)					
Never drink	564,107(36.9)	414,963(35.7)	126,424(40.3)	22,720(44.9)	128,129(46.3)
Current drinkers	437,119(28.6)	284,955(24.5)	128,561(40.9)	23,603(46.6)	127,972(46.3)
Past drinkers	6727(0.4)	4655(0.4)	1730(0.6)	342(0.7)	1,534(0.6)
Unknown	518,733(34.0)	457,470(39.4)	57,319(18.3)	3944(7.8)	18,873(6.8)

^a^ Non-Han ethnicities include Tibetan, Hui, Yi, Tujia, Man, Qiang, Miao, Menggu, Bai, Zhuang, Buyi, Dong, Qilao, Chaoxian, Yao, Naxi, Hani, Dai, Xibo, Shui, Jing, Yaolao, Weiwuer, Dahaner, Li, Susu, Pumi, Elunchun, Lagu, Achang, Hasake, Menba, Gaoshan, Keerkezi, Sala, and Yugu

^b^ Other occupation include military personnel, actors, freelancers

### Changes in health metrics or biomarkers with ageing

**Fig. 5:**
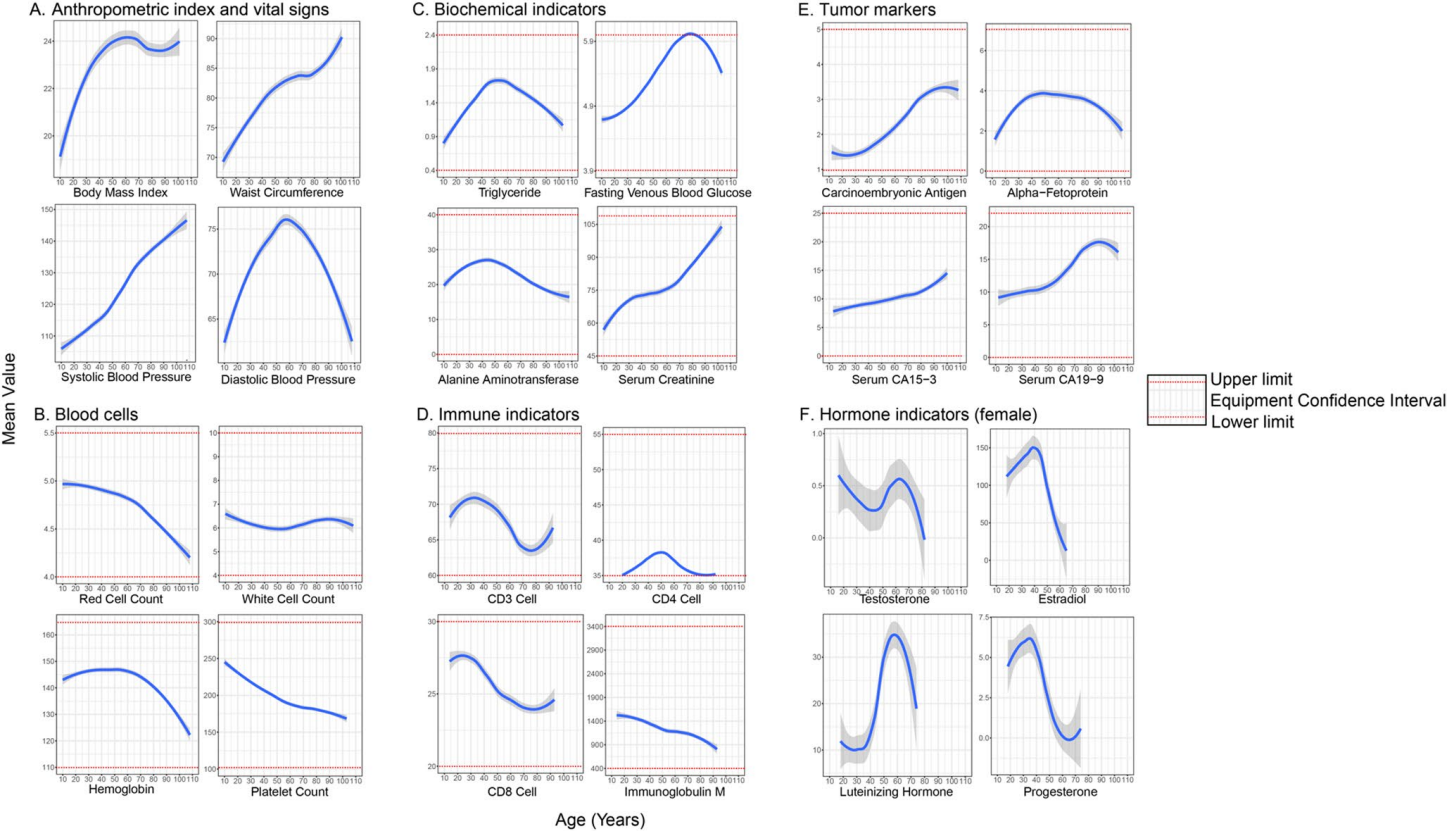
Trajectories of health metrics or biomarkers along with ageing

To investigate how specific health metrics and biomarkers (i.e., anthropometric and vital sign, blood cell counts, biochemical indices, immunity-related markers, tumor markers, and hormonal levels) vary with age, we plotted their values across different age groups (Fig. 5). Overall, all examined indicators exhibited age-related changes, albeit with distinct patterns. For example, waist circumference, systolic blood pressure, and serum creatinine showed a monotonic increase with age, while red blood cell count, platelet count, and immunoglobulin M levels displayed a linear decline with age. In contrast, some indicators, particularly sex hormones, followed a more complex wave-like pattern. Notably, fasting venous blood glucose levels approached the upper limit of the normal range in individuals aged 70–90 years, whereas CD4 cell counts neared the lower limit in the same age group. These findings suggest the potential need for age-specific adjustments to normal reference ranges for certain biomarkers. In summary, monitoring these metrics across age groups provides valuable insights into the natural progression of physiological changes associated with aging and highlights the importance of tailoring reference ranges to reflect age-specific variations.

### Associations of health metrics with pre-disease conditions and diseases

**Fig. 6:**
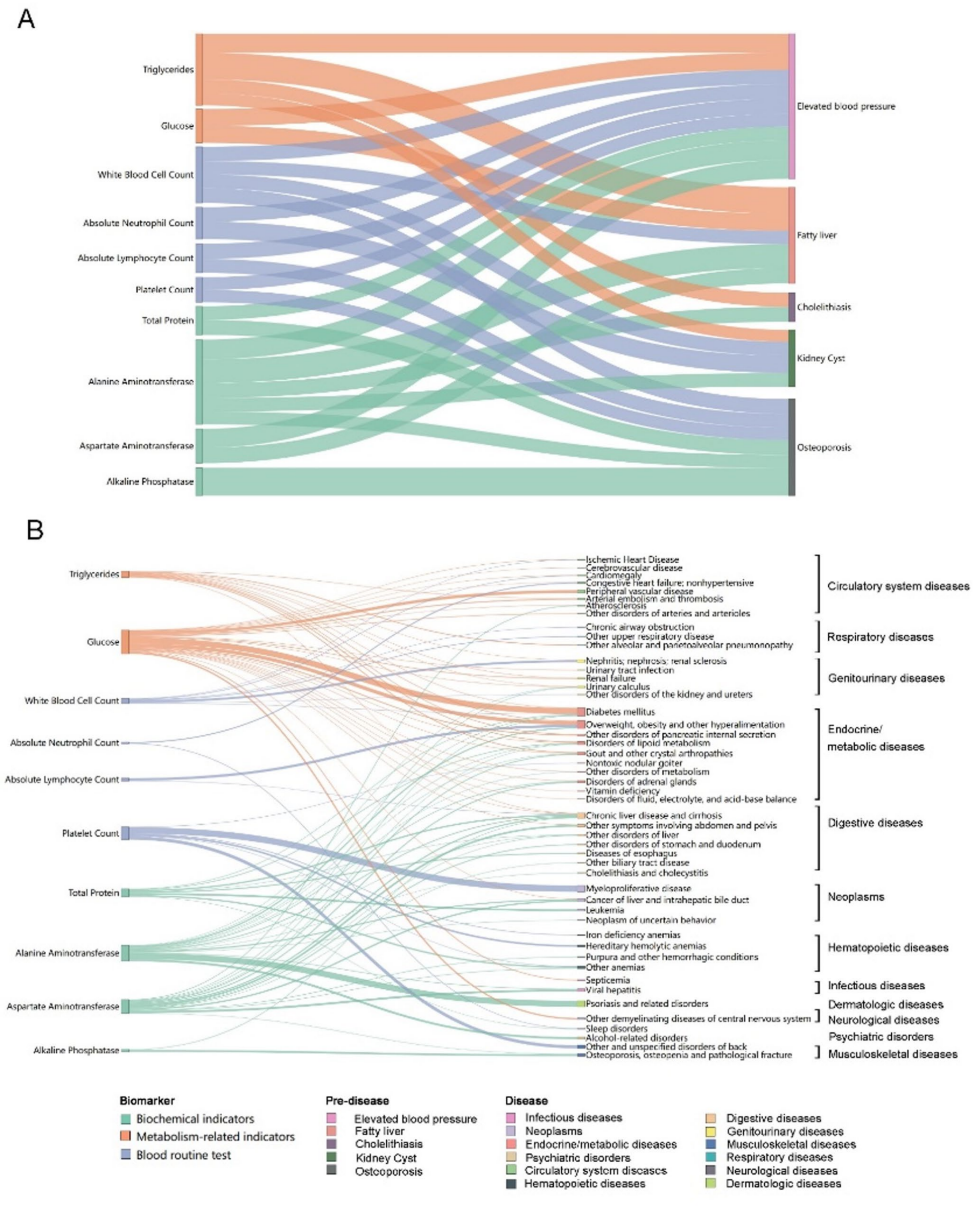
Associations of elevated health metrics with pre-disease conditions and disease diagnoses. We used odds ratios (ORs) obtained from conditional logistic regression to quantify the strength of associations, with thicker lines representing stronger associations. Panel (**A**) illustrates the relationships between elevated biomarkers and pre-disease states, while Panel (**B**) depicts the associations between elevated biomarkers and inpatient diagnoses (all diagnosis codes were mapped to “Phecodes”, a coding system more aligned with clinically relevant diseases). Sex-specific pre-diseases and diseases were excluded from the analysis. All displayed lines represent statistically significant ORs greater than 1 (without multiple testing correction). The connections between biomarkers and pre-disease states, as well as those between pre-disease and disease states, were calculated independently

Understanding the relationship of health metrics with pre-disease conditions and disease diagnoses is crucial for identifying early markers of health deterioration and potential intervention points. Using a matched-cohort design, we analyzed the associations between abnormalities in biomarkers—such as metabolism-related and inflammatory indicators—and the presence of pre-disease conditions (e.g., elevated blood pressure [diastolic blood pressure > 85 mmHg or systolic blood pressure > 130 mmHg, appearing twice in health check records], fatty liver, and cholelithiasis [according to Doppler ultrasound findings]) or clinical diagnoses of various diseases. Relative risks were estimated using conditional Logistic Regression models (Fig. 6). For each exposed individual, one matched unexposed individuals were randomly selected from the whole study population, individually matched by sex and birth year. Additionally, a temporal constraint was applied, requiring that the control subject’s last follow-up date before the exposed subject’s index date (i.e., the date of the first exposure event). In the conditional logistic regression model, the matched groups were used as the stratum variable. The model was further adjusted for marital status and educational attainment. Our findings revealed clear yet complex connections among these three stages of health. For example, elevated triglyceride levels were linked to an increased risk of multiple pre-disease conditions, as well as a wide range of cross-system disease diagnoses. By mapping these association networks, we can gain deeper insights into disease progression, enhance early risk identification, and refine strategies for targeted prevention and management before clinical symptoms emerge.

### Identification of individual-level health trajectory patterns and their association with various diseases

**Fig. 7:**
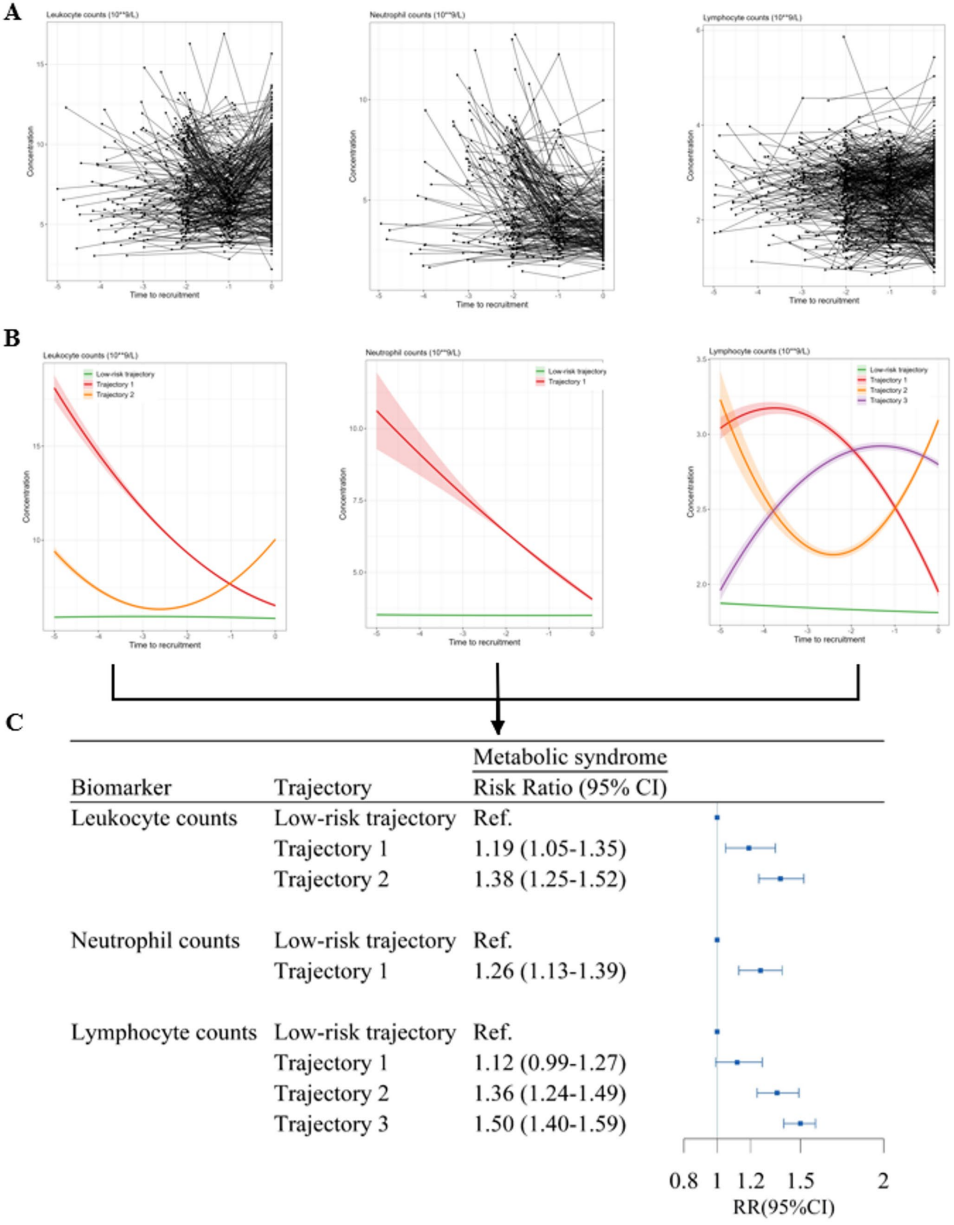
Individual-level trajectories of inflammatory biomarkers and the association with metabolic syndrome. **A** the longitudinal trends of inflammatory biomarkers for a representative sample of 100 participants selected from the full dataset. Each line represents an individual participant, with points indicating observed values at multiple time points. **B** Individual-level trajectories of inflammatory biomarkers using latent class mixed models. The lines with the shadows represent mean concentrations and 95% confidence intervals of inflammatory biomarkers for each latent class of individuals following similar change patterns over time. **C** The associations between Individual-level trajectories of inflammatory biomarkers and metabolic syndrome. Risk Ratio and 95% CIs were estimated by Poisson regression with robust error variance, adjusting for sex, age at first blood sampling, year of examination, smoking status, and drinking status, education level, income level and a follow-up time offset

Using data from the WHALE Health Trajectory Cohort, we conducted studies examining the associations between trajectories of inflammatory biomarkers and the development of metabolic syndrome and chronic kidney diseases. This work focuses on identifying significant patterns in individual-level biomarker trajectories that precede the onset of diseases or symptoms using latent class mixed models with second-order polynomials. For instance, among participants with at least three measurements of leukocyte, neutrophil, and lymphocyte counts within five years prior to enrollment, we identified latent classes representing individuals with distinct trajectory patterns over time (Fig. 7A, B). The results of association analyses indicated that notable changes in white blood cell counts often occurred before the disease onset (e.g., metabolic syndrome, Fig. 7C), highlighting the role of inflammatory pathways in the pathogenesis of those diseases.

### Other publications and ongoing projects

A recent study developed the Chinese Lung Nodules Reporting and Data System (C-Lung-RADS) using 45,064 cases from WHALE, integrating imaging, demographic, and follow-up data to enhance risk stratification and precision management of pulmonary nodules. This system significantly improved early lung cancer detection and reduced unnecessary invasive procedures, demonstrating the value of large-scale, real-world health checkup data in advancing personalized screening and management strategies [25]. Using the first release of the WHALE Database, which includes 478,898 individuals, we identified 998 COVID-19 cases in December 2022 (i.e., one month following the relaxation of policies in China) utilizing linked clinical records [15]. Our analysis revealed significant associations between the severity of COVID-19 and various baseline hematological parameters, along with their trajectories prior to infection onset. Specifically, elevated basophil and monocyte percentages, as well as abnormalities in red cell distribution width (RDW) and mean corpuscular hemoglobin concentration, were associated with more severe COVID-19 outcomes. Additionally, trajectory patterns of RDW and certain white blood cell counts were significantly associated with an increased risk of severe COVID-19, highlighting the potential value of these parameters for preemptive health assessments.

## Discussion

### WHALE study in comparison with other studies

The WHALE Study stands out among global and Chinese cohort studies for its frequent longitudinal health assessments and integration within a standardized hospital-based health network (Supplementary Table 6). Among domestic cohorts in China, WHALE further distinguishes itself through its repeated, multimodal assessments embedded in routine clinical care. Compared with China Kadoorie Biobank (CKB, ~ 512,000 participants) [26], which primarily focuses on genetic and chronic disease risk factors across multiple regions, WHALE emphasizes frequent longitudinal health assessments, offering richer imaging and laboratory data through extensive repeated check-ups integrated within a hospital-based health system. Relative to the Kailuan Study (~ 100,000 participants) [27], which centers on cardiovascular and occupational health among industrial workers in Tangshan, WHALE provides a larger and more diverse sample with broader disease coverage, benefiting from repeated multimodal health assessments and a comprehensive hospital-based data collection network. In contrast with the Taizhou Longitudinal Study (~ 200,000 participants) [28], oriented mainly towards genetic and environmental research in Jiangsu province, WHALE uniquely combines biennial active follow-ups and detailed multimodal data, leveraging repeated measurements across physical, laboratory, and imaging domains integrated systematically through hospital-based infrastructures.

### Strengths

The WHALE Study demonstrates distinct advantages among Chinese and global cohorts through its integration of frequent longitudinal assessments, comprehensive individual-level data, and standardized hospital-based infrastructure. These features jointly enable refined trajectory modeling and enhance translational relevance. Specifically, WHALE’s Health Trajectory Cohort requires a minimum of three health check-ups, resulting in 23.88% of participants having more than three visits, with 3.31% exceeding ten visits. This high frequency of repeated measurements enables robust longitudinal analyses, facilitating the identification of detailed individual-level health trajectories (e.g., biomarkers, imaging) and health-to-disease transitions through advanced statistical approaches. Additionally, WHALE study leverages integration within the West-China Hospital Alliance, comprising 11 hospitals across Sichuan Province. This alliance ensures standardized multidimensional data collection—covering physical examinations, laboratory tests, imaging, questionnaires, and biospecimens—and rigorous quality control using the Plan-Do-Check-Act framework. Furthermore, WHALE Study’s longitudinal design integrates biennial active follow-ups (via automated reminders and nurse-led contacts) and semi-annual passive linkage to regional health databases, minimizing attrition and supporting continuous and comprehensive outcome tracking.

### Weaknesses

First, the healthcare-related nature of this database may introduce certain limitations. For instance, the WHALE Study’s reliance on organization-based or employer-mandated health check-ups may introduce selection bias by overrepresenting younger, occupationally active, or health-conscious individuals, potentially limiting generalizability to broader populations, including older adults, rural residents, low-income groups, or those with limited healthcare engagement. This bias may affect the external validity of our findings, particularly for underserved populations. However, the study’s large sample size, ethnic diversity, and coverage of 11 hospitals across urban, rural, and high-altitude regions in Sichuan Province partially mitigate this concern by capturing a wide range of socioeconomic and environmental backgrounds.

More importantly, WHALE Study incorporated strategies to assess and mitigate its potential impact, both at the design and analysis levels. At the design level, the cohort includes participants from both self-initiated and employer-mandated screenings to capture diverse health profiles. The West-China Hospital Alliance, spanning 11 hospitals across urban centers and rural/high-altitude regions in Sichuan Province, enhances inclusiveness by covering varied geographic and socioeconomic populations. Additionally, ongoing collaborations with community-based health service centers aim to expand the network beyond tertiary hospitals, reaching populations with differing healthcare access. At the analysis level, we compared key baseline characteristics between the Health Trajectory Cohort and the broader WHALE database population (Table 1), confirming broad comparability. Multivariable-adjusted models and matched cohort designs were employed to reduce confounding, and inverse probability weighting (IPW) is being applied in ongoing analyses to further adjust for selection bias, with sensitivity analyses to evaluate result robustness.

Second, the requirement for participants to have at least three health check-ups introduces the potential for survivor bias. Individuals who remained engaged in follow-up may differ systematically from those lost to follow-up in terms of health status, socioeconomic background, or health behaviors. This could affect the generalizability of our findings and may lead to an underestimation of early event risks. To address this, future analyses will incorporate sensitivity analyses and methods such as inverse probability weighting to evaluate and mitigate the impact of this potential bias.

Third, self-reported data on lifestyle (e.g., smoking, diet, physical activity) and psychological measures (e.g., anxiety, depression) may introduce recall and reporting biases, potentially affecting data accuracy. To mitigate this, we used validated tools like the PHQ-9, GAD-7, and IPAQ short form, cross-validated self-reported medical history with clinical records for over 510,000 participants, and conducted reliability checks, including test-retest assessments and consistency evaluations across repeated check-ups. Future improvements include integrating wearable devices for objective lifestyle data and AI-based tools for real-time validation to further enhance data quality.

Finally, a significant challenge for the Health Trajectory Cohort is the risk of loss to follow-up, particularly among participants recruited during the historical phase. Since these participants may have completed their initial check-ups years ago without the active follow-up protocols in place, there is a greater chance they may not return for ongoing monitoring. However, such a concern could be somehow alleviated by the availability of linked data from regional healthcare and administrative records, which help supplement long-term health outcomes for all cohort participants, maintaining data effectiveness for longitudinal analyses.

### Generalizability of findings

The WHALE Study shows strong generalizability at both the institutional and regional levels. At the institutional level, it is anchored at West China Hospital, a top national medical center serving over 80 million people, with data from 11 affiliated hospitals using standardized protocols. This hospital-based design enables systematic capture of longitudinal clinical records and ensures consistent follow-up and biomarker integration—features often challenging in community-based cohorts. At the regional level, Sichuan Province offers rich demographic and ecological diversity, including urban and high-altitude rural areas, and multiple ethnic groups. These features enhance the study’s relevance for understanding health-to-disease transitions in diverse Chinese populations. However, generalizability to other parts of China or global populations may be limited by contextual differences, such as healthcare infrastructure and environmental exposures. Despite this, the study’s hospital-based design, large sample size (1.5 million), longitudinal follow-up, and multidimensional data provide a solid foundation for comparative analyses and future translational research.

### Potential for future studies

The WHALE dataset captures 1,083 longitudinal metrics from exams, labs, imaging, genomics and questionnaires; 23.88% of participants have ≥ 3 check-ups linked to ICD-10 records and biospecimens. Ongoing work will (i) model biomarker trajectories to predict cardiometabolic, oncologic, neuropsychiatric and infectious outcomes; (ii) examine bidirectional links between mental-health status and biological ageing, testing lifestyle and socioeconomic mediators; and (iii) map health-disease pathways using geocoded urban-rural exposures (e.g. air pollution). Planned expansions in metabolomics, proteomics and wearables, coupled with AI-driven risk scores, will support precision prevention and inform environment- and mental-health policy.

The WHALE Study supports diverse research and welcomes external collaboration. Data access is reviewed by the WHALE Data Access Committee based on scientific merit and ethical compliance, with approved users accessing de-identified data in a secure environment. A dedicated website is being developed to share application guidance and results. Inquiries are welcome via email (Dr. Yifei Lin: ylin@wchscu.edu.cn).

## Supplementary Information

Below is the link to the electronic supplementary material.

Supplementary Material 1

## References

[1] Murray CJL. The global burden of disease study at 30 years. Nat Med. 2022;28(10):2019–26. 36216939 10.1038/s41591-022-01990-1

[2] Liu J, Li W, Yao H, Liu J. Proactive health: an imperative to achieve the goal of Healthy China. China CDC Wkly. 2022. 10.46234/ccdcw2022.156. 36285279 10.46234/ccdcw2022.156 PMC9547729

[3] Wu S, An S, Li W, et al. Association of trajectory of cardiovascular health score and incident cardiovascular disease. JAMA Netw Open. 2019;2(5):e194758–194758. 31150075 10.1001/jamanetworkopen.2019.4758 PMC6547110

[4] Alpert A, Pickman Y, Leipold M, et al. A clinically meaningful metric of immune age derived from high-dimensional longitudinal monitoring. Nat Med. 2019;25(3):487–95. 30842675 10.1038/s41591-019-0381-y PMC6686855

[5] Daskalopoulou C, Koukounari A, Wu YT, et al. Healthy ageing trajectories and lifestyle behaviour: the Mexican health and aging study. Sci Rep. 2019;9(1):11041. 31363117 10.1038/s41598-019-47238-w PMC6667468

[6] GBD 2019 Universal Health Coverage Collaborators. Measuring universal health coverage based on an index of effective coverage of health services in 204 countries and territories, 1990–2019: a systematic analysis for the global burden of disease study 2019. Lancet. 2020;396(10258):1250–84. 32861314 10.1016/S0140-6736(20)30750-9 PMC7562819

[7] Mirza SS, Wolters FJ, Swanson SA, et al. 10-year trajectories of depressive symptoms and risk of dementia: a population-based study. Lancet Psychiatry. 2016;3(7):628–35. 27138970 10.1016/S2215-0366(16)00097-3

[8] Grimes PZ, Adams MJ, Thng G, et al. Genetic architectures of adolescent depression trajectories in 2 longitudinal population cohorts. JAMA Psychiatr. 2024;81(8):807. 10.1001/jamapsychiatry.2024.0983 PMC11097103 38748406

[9] Zeng Y, Chourpiliadis C, Hammar N, et al. Inflammatory biomarkers and risk of psychiatric disorders. JAMA Psychiatr. 2024. 10.1001/jamapsychiatry.2024.2185. 10.1001/jamapsychiatry.2024.2185 PMC11339698 39167384

[10] Hozawa A, Tanno K, Nakaya N, et al. Study profile of the Tohoku medical megabank Community-Based cohort study. J Epidemiol. 2021;31(1):65–76. 31932529 10.2188/jea.JE20190271 PMC7738642

[11] Walldius G, Malmström H, Jungner I, et al. Cohort profile: the AMORIS cohort. Int J Epidemiol. 2017;46(4):1103–1103i. 28158674 10.1093/ije/dyw333

[12] Gong W, Cheng KK. Challenges in screening and general health checks in China. Lancet Public Health. 2022;7(12):e989-90. 36462520 10.1016/S2468-2667(22)00207-9

[13] Meinian Onehealth Healthcare Holdings Co., Ltd. Summary of the 2024 annual report [Internet]. 2024 [cited 2025 Aug 13]. Available from: https://static.cninfo.com.cn/finalpage/2025-04-17/1223113459.PDF

[14] Universtiy WCHoS. West-China Hospital Alliance. 2012. https://www.wchscu.cn/ylt.html. Accessed 8 Jul 2025.

[15] Lin Y, Yang Y, Xiang N, et al. Characterization and trajectories of hematological parameters prior to severe COVID-19 based on a large-scale prospective health checkup cohort in Western China: a longitudinal study of 13-year follow-up. BMC Med. 2024;22(1):105. 38454462 10.1186/s12916-024-03326-x PMC10921814

[16] Wang M, Shi K, Chen L et al. Analysis of the features of lead-type close medical consortium in West China Hospital of Sichuan University. West China Medical Journal. 2019;34(12):1422–1425.

[17] Wang M, Li S, Zheng T, et al. Big data health care platform with multisource heterogeneous data integration and massive high-dimensional data governance for large hospitals: design, development, and application. JMIR Med Inf. 2022;10(4):e36481. 10.2196/36481 PMC9047713 35416792

[18] Bingjie Q, Jing Z, Ying D, Ting D. Analysis on the causes of death and potential life loss of malignant tumors in Sichuan Province from 2017 to 2022. China Cancer. 2025;34(01):37–42.

[19] Kun T, Wen C, Jing-ping P, et al. Analysis on medical record first page data quality from Sichuan health statistics reporting system. Soft Sci Heath. 2015;29(10):637–41.

[20] Wang W, Bian Q, Zhao Y, et al. Reliability and validity of the Chinese version of the patient health questionnaire (PHQ-9) in the general population. Gen Hosp Psychiatry. 2014;36(5):539–44. 25023953 10.1016/j.genhosppsych.2014.05.021

[21] Zhang C, Wang T, Zeng P, et al. Reliability, validity, and measurement invariance of the general anxiety disorder scale among Chinese medical university students. Front Psychiatry. 2021;12:648755. 34093269 10.3389/fpsyt.2021.648755 PMC8170102

[22] Wang C, Chen P, Zhuang J. Validity and reliability of international physical activity questionnaire–short form in Chinese youth. Res Q Exerc Sport. 2013;84(sup2):S80-6. 24527570 10.1080/02701367.2013.850991

[23] Fan P, Zhang S, Wang W, et al. The design and implementation of natural population cohort study biobank: a multiple-center project cooperation with medical consortia in Southwest China. Front Public Health. 2022;10:996169. 36530701 10.3389/fpubh.2022.996169 PMC9751194

[24] Realyvásquez Vargas A, García Alcaraz JL, Satapathy S, Coraza DA, Báez López Y. The PDCA (Plan-Do-Check-Act) cycle. In: García Alcaraz JL, Robles GC, Realyvásquez Vargas A, editors. Lean manufacturing in Latin america: concepts, methodologies and applications. Cham: Springer Nature Switzerland; 2025. pp. 409–37.

[25] Wang C, Shao J, He Y, et al. Data-driven risk stratification and precision management of pulmonary nodules detected on chest computed tomography. Nat Med. 2024;30(11):3184–95. 39289570 10.1038/s41591-024-03211-3 PMC11564084

[26] Chen Z, Chen J, Collins R, et al. China kadoorie biobank of 0.5 million people: survey methods, baseline characteristics and long-term follow-up. Int J Epidemiol. 2011;40(6):1652–66. 22158673 10.1093/ije/dyr120 PMC3235021

[27] Liu X, Cui L, Wang A, et al. Cumulative exposure to ideal cardiovascular health and incident diabetes in a Chinese population: the Kailuan study. J Am Heart Assoc. 2016;5(9):e004132. 27638783 10.1161/JAHA.116.004132 PMC5079052

[28] Wang X, Lu M, Qian J, et al. Rationales, design and recruitment of the Taizhou longitudinal study. BMC Public Health. 2009;9(1):223. 19589173 10.1186/1471-2458-9-223 PMC2715397

