# Supplementary material for: Cohort profile: the West-China hospital alliance longitudinal epidemiology wellness (WHALE) study
Source: Eur J Epidemiol. 2025 Aug 23;40(9):1143–59. doi: 10.1007/s10654-025-01290-1 (PMC12537590; doi:10.1007/s10654-025-01290-1)
Supplement: Supplementary file 1 — Supplementary Material 1 [file 10654_2025_1290_MOESM1_ESM.docx]

**Cohort Profile: The West-China Hospital Alliance Longitudinal Epidemiology Wellness (WHALE) Study**

**Supplementary Materials**

Supplementary Table 1. The timeline of data integrating in the WHALE Database

Supplementary Figure 1. Quality control process of data collection

Supplementary Table 2 The Number of New Participants in Different Years and Their Frequency of Health Check-ups by November 5, 2024

Supplementary Table 3. List of characteristics measured among health check-up in the WHALE

Supplementary Table 4. Health Examination Equipment Information

Supplementary Table 5. Measurement tools in questionnaires

Supplementary Figure 2. The volume range of plasma in each sample tube

Supplementary Figure 3. Flowchart of data clean

Supplementary Table 6. Comparative summary table contrasting WHALE with other major cohorts

## Supplementary Table 1. The timeline of data integrating in the WHALE Database (As of Jan 15, 2025)

| Order | Hospitals in WHALE database | Enrollment Time | Integration  Sequence | Participants | Location | Hospital Level |
| --- | --- | --- | --- | --- | --- | --- |
| 1 | West China Hospital, Sichuan University | 2010.01 | Tier-1 | 571294 | Chengdu, Sichuan | Tertiary |
| 2 | West China Hospital of Sichuan University (Wenjiang campus) | 2013.07 | Tier-1 | 311096 | Chengdu, Sichuan | Tertiary |
| 3 | Shang Jin Hospital of West China Hospital, Sichuan University | 2016.03 | Tier-1 | 63714 | Chengdu, Sichuan | Tertiary |
| 4 | Wuhou Health Examination Center of West China Hospital | 2019.10 | Tier-1 | 149554 | Chengdu, Sichuan | Health Management Center |
| 5 | Tian Fu Hospital of West China Hospital, Sichuan University | 2021.10 | Tier-1 | 98932 | Chengdu, Sichuan | Tertiary |
| 6 | The First People’s Hospital of Shuangliu District, Chengdu | 2024.01 | Tier-2 | 29484 | Chengdu, Sichuan | Tertiary |
| 7 | Meishan People's Hospital, Meishan | 2024.04 | Tier-2 | 2331 | Meishan, Sichuan | Tertiary |
| 8 | Mianzhu People's Hospital, Mianzhu | 2024.04 | Tier-2 | 125997 | Mianzhu, Sichuan | Tertiary |
| 9 | Jintang County First People's Hospital, Chengdu | 2024.05 | Tier-2 | 87832 | Chengdu, Sichuan | Tertiary |
| 10 | Wangjiang Hospital, Sichuan University | 2024.05 | Tier-2 | 168778 | Chengdu, Sichuan | Secondary |
| 11 | West China Hospital, Sichuan University (Jinjiang campus) | 2025.02 | Tier-1 | Opening in 2025.02 | Chengdu, Sichuan | Tertiary |

## Supplementary Figure 1. Quality control process of data collection


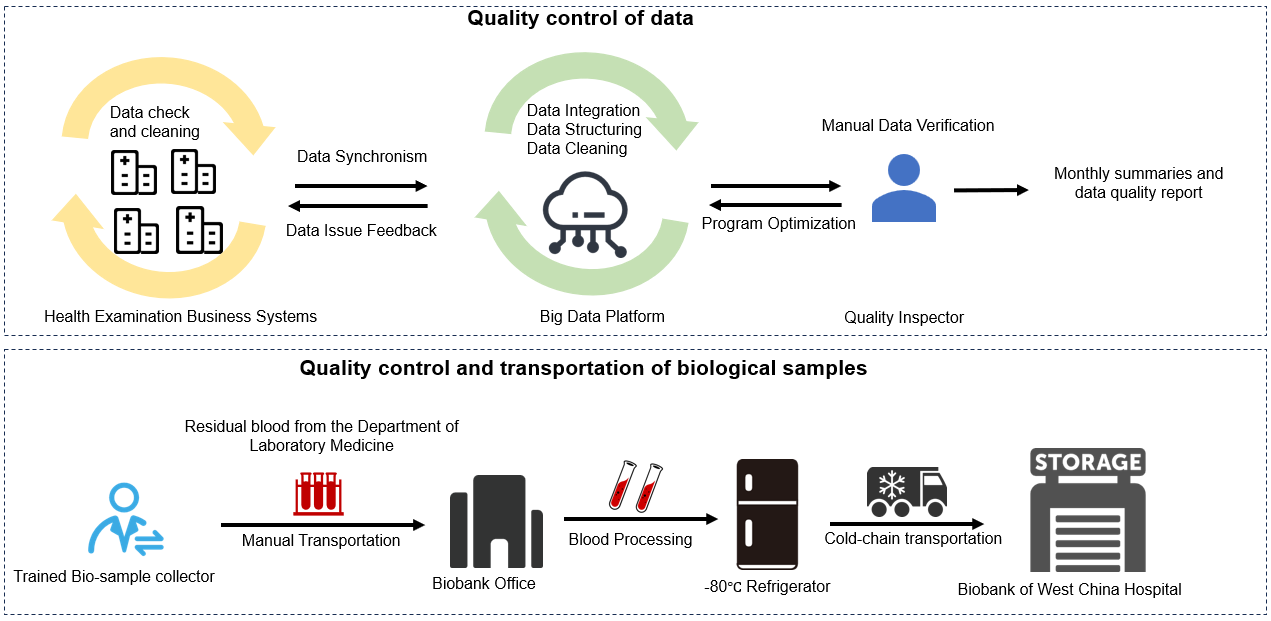


## Supplementary Table 2. The number of new participants in different years and their frequency of health check-ups by January 15^th^, 2025

| Year | New participants | The Number of Individuals with Different Frequencies of Health Check-ups as of January 15***^th^***, 2025 | | | | | | | | | |
| --- | --- | --- | --- | --- | --- | --- | --- | --- | --- | --- | --- |
|  |  | **1** | **2** | **3** | **4** | **5** | **6** | **7** | **8** | **9** | **≥10** |
| 2010 | 70,485 | 22,932 | 7,120 | 4,198 | 3,689 | 2,873 | 3,089 | 3,123 | 2,540 | 1,952 | 18,969 |
| 2011 | 53,877 | 20,392 | 6,572 | 3,559 | 2,816 | 2,931 | 2,766 | 2,693 | 1,721 | 1,467 | 8,960 |
| 2012 | 51,605 | 23,965 | 5,686 | 3,181 | 2,933 | 2,656 | 1,632 | 1,098 | 1,120 | 1,228 | 8,106 |
| 2013 | 54,819 | 24,246 | 6,918 | 4,926 | 5,230 | 2,833 | 1,919 | 1,851 | 1,201 | 1,234 | 4,461 |
| 2014 | 75,904 | 33,377 | 10,643 | 7,532 | 3,919 | 3,269 | 2,804 | 2,574 | 2,418 | 2,409 | 6,959 |
| 2015 | 64,698 | 30,287 | 10,346 | 5,440 | 3,905 | 2,929 | 2,336 | 2,017 | 2,009 | 2,565 | 2,864 |
| 2016 | 71,125 | 39,122 | 9,493 | 5,260 | 3,820 | 2,570 | 2,089 | 1,924 | 2,687 | 4,002 | 158 |
| 2017 | 86,650 | 46,557 | 12,081 | 8,096 | 3,938 | 3,687 | 3,497 | 3,854 | 4,677 | 211 | 52 |
| 2018 | 106,286 | 61,068 | 16,376 | 7,757 | 5,335 | 4,506 | 5,848 | 4,945 | 255 | 149 | 47 |
| 2019 | 118,777 | 77,582 | 17,244 | 7,089 | 4,830 | 6,201 | 5,580 | 194 | 26 | 11 | 20 |
| 2020 | 143,534 | 87,669 | 19,881 | 11,629 | 1,5934 | 8,148 | 205 | 35 | 13 | 12 | 8 |
| 2021 | 163,095 | 103,661 | 23,756 | 23,782 | 1,1513 | 320 | 46 | 11 | 3 | 1 | 2 |
| 2022 | 176,317 | 112,717 | 43,875 | 19,294 | 358 | 53 | 11 | 2 | 3 | 2 | 2 |
| 2023 | 205,188 | 173,449 | 30,733 | 931 | 56 | 9 | 4 | 3 | 2 |  | 1 |
| 2024 | 83354 | 82,683 | 640 | 24 | 5 | 1 |  |  | 1 |  |  |
| 2025 | 972 | 972 |  |  |  |  |  |  |  |  |  |
| Total | 1,526,686 | 940,679 | 221,364 | 112,698 | 68,281 | 42,986 | 31,826 | 24,324 | 18,676 | 15,243 | 50,609 |

## Supplementary Table 3. List of characteristics measured among health check-up in the WHALE.

|  | Categories | Available information |
| --- | --- | --- |
| Demographics | Demographics | National ID number, Unique ID, Birthdate, Gender, Marital Status, Ethnic Group, Education, Occupation, Corporate Name, Contact Information, Physical Examination Organization |
| Face-to-Face interview | Health related information | Smoking Status, Alcohol Consumption, Diseases History, Family History, Surgical History, Drug Allergy History |
| Physical examination | Anthropometry | Body Weight, Body Height, Waist Circumference, Hip Circumference, Body Mass Index (BMI) |
|  | Specialist examination | Internal Checkup: Liver, Kidney, Spleen, Lung, Heart; External Checkup: Skin, Lymph Gland, Spine, Breast, Thyroid, Apparatus Urogenitalis; Ophthalmic Testing: Eye Ground, Eyesight, Intraocular Pressure, Eyeball, Pupil, Eyelid; Otorhinolaryngology Examination: Aural Region, Nose, Oropharynx, Tonsil, Laryngeal; Oral, Gynecologic Examination: Vagina, Uterine Neck, Vulva, Uterus, Secreta |
|  | Other Specialist examination | Bone Density Test, Bone Metabolism Test, Fatty Liver and Liver Fibrosis Assessment, Body Composition Analysis, Pulmonary Function Test，Electrocardiogram (ECG) |
| Laboratory tests | Hematology | Hematocrit, Red Blood Cell Distribution Width - Standard Deviation (RDW-SD), Mean Corpuscular Hemoglobin (MCH), Mean Corpuscular Hemoglobin Concentration (MCHC), Mean Corpuscular Volume (MCV), White Blood Cell Count (WBC), Hemoglobin (Hb), Red Blood Cell Count (RBC), Platelet Count, Red Blood Cell Distribution Width - Coefficient of Variation (RDW-CV), Neutrophil Percentage, Lymphocyte Percentage, Lymphocyte Absolute Count, Neutrophil Absolute Count, Monocyte Percentage, Monocyte Absolute Count, Eosinophil Percentage, Eosinophil Absolute Count, Basophil Percentage, Basophil Absolute Count |
|  | Urine Test | Urine color, Urine turbidity, Urine glucose, Qualitative urine bilirubin, Qualitative ketone bodies, Nitrite, Urine occult blood, Urine white blood cells, Percentage of lysed red blood cells, Absolute count of lysed red blood cells, Amorphous phosphate crystals, Calcium oxalate crystals, Urobilinogen Qualitative Test, Specific Gravity, pH Value, Casts, Bacteria, Urine Protein Qualitative Test, Urine Microalbumin |
|  | Stool Test | Consistency, Color, Occult Blood, Microscopic White Blood Cells, Red Blood Cells., Phagocytes, Starch Granules, Fat Droplets, Yeast-Like Organisms |
|  | Liver Function Tests | Aspartate Aminotransferase (AST), Alanine Aminotransferase (ALT), AST/ALT Ratio, Indirect Bilirubin, Total Bilirubin, Alkaline Phosphatase (ALP), Albumin/Globulin Ratio, Total Protein, Direct Bilirubin, Globulin, Albumin, Gamma-Glutamyl Transferase (GGT) |
|  | Kidney Function | Creatinine, Estimated Glomerular Filtration Rate (eGFR), Urea, Uric Acid, blood urea nitrogen, Cystatin C |
|  | Glucose Metabolism | Glucose, Hemoglobin A1c (HbA1c), Glycated Albumin, Fasting Insulin, Fasting C-Peptide |
|  | Thyroid function Tests | Triiodothyronine, Free Thyroxine, Free Triiodothyronine, Thyroid Stimulating Hormone Receptor Antibodies, Thyroglobulin Antibodies, Thyroglobulin, Thyroxine, Thyroid Stimulating Hormone, Reverse Triiodothyronine, Thyroid Peroxidase Antibodies, Parathyroid Hormone, Sensitive Thyroid Stimulating Hormone |
|  | Tumor Markers | Carcinoembryonic Antigen (CEA), Alpha-Fetoprotein (AFP), Prostate-Specific Antigen (PSA), Carbohydrate Antigen 19-9, Carbohydrate Antigen 15-3, Cancer Antigen 125, CYFRA21-1 |
|  | Lipid Profile | Triglycerides, Total Cholesterol, High-Density Lipoprotein (HDL), Low-Density Lipoprotein (LDL) |
|  | Infectious Diseases | Human papilloma virus (HPV), SARS-CoV-2 ORF1ab Gene Nucleic Acid, SARS-CoV-2 N Gene Nucleic Acid, Hepatitis C Virus Antibody, Hepatitis B Surface Antigen (HBsAg), Hepatitis B e-Antigen (HBeAg), Hepatitis B Core Antibody (HBcAb), Hepatitis B Surface Antibody (HBsAb), Cytomegalovirus Antibody IgG, Cytomegalovirus Antibody IgM, HIV Antibodies (I/II) |
|  | Inflammation and Immunology | C-Reactive Protein (CRP), Erythrocyte Sedimentation Rate (ESR), Anti-Nuclear Antibody (ANA), Rheumatoid Factor (RF), Immunoglobulin M (IgM), Immunoglobulin G (IgG), Immunoglobulin A (IgA), Immunoglobulin E (IgE) |
|  | Hormones | Testosterone, Estradiol, Progesterone, Follicle Stimulating Hormone (FSH), Luteinizing Hormone (LH), Prolactin (PRL), Gonadotropins, Prolactin, Insulin, Insulin-like Growth Factor 1 (IGF-1), Dopamine, Norepinephrine, Growth Hormone (GH) |
|  | Gastrointestinal System | Pepsinogen I (PG I), Pepsinogen II (PG II), Pepsinogen I/II Ratio, Gastrin, Helicobacter pylori Urease Antibody, Amylase, Lipase |
|  | Electrolytes and Minerals | Sodium, Potassium, Carbon Dioxide Combining Power, Phosphorus, Chloride, Anion Gap, Calcium, Phosphorus, Magnesium |
|  | Other Biochemical Tests | Lactate Dehydrogenase (LDH), Hydroxybutyrate Dehydrogenase (HBDH), Creatine Kinase (CK), 25-Hydroxy Vitamin D |
|  | Gynecologic examination | Candida in Vaginal Discharge, Cleanliness of Vaginal Discharge, Leukocyte Esterase, Hydrogen Peroxide, Fungi, Lactobacillus, Candida, Proline Aminopeptidase, pH of Vaginal Discharge |
| Image tests | CT | Chest, Whole Abdomen, Upper Abdomen, Head, Neck, Pelvis, Spine |
|  | X-ray | Cervical Spine, Thoracic Spine, Lumbar Spine, Extremity Bones, Chest, Abdominal Plain Film, Gastrointestinal Series |
|  | Ultrasonography | Liver, Gallbladder, Pancreas, Spleen, Kidney, Adrenal Gland, Thyroid Gland, Breast, Lymph Node |

## Supplementary Table 4. Health examination equipment information

| **health check-up type** | **Examination items** | **Equipment Model and Version** | **Company** |
| --- | --- | --- | --- |
| Physical examination | Blood Pressure | ABP-1000(F-version） | Chioy, China |
|  | Standing Height/ Weight | SG-1001SC | Chioy, China |
|  |  | SK-V7 | Sonka, China |
|  | Bone density measurements | MetriScan | Miles Medical LLC, China |
|  | Pulmonary function testing | MasterScreen SeS | Jaeger, Germany |
|  | Body composition analysis | InBody570 | InBody, Korea |
|  | Liver Fibrosis Determination | FibroScan 502 Touch | ECHOSENS, France |
|  |  | FT-C | , China |
|  |  | Hepatus 6 | Mindray, China |
|  | Slit-lamp Microscope | SL-2G/DKT-18 | TOPCON, Japan |
|  |  | YZ5J | 66 Vision-Tech |
|  |  | SLM-5E | KANGHUA, China |
|  | Fundus Camera | CR-2 AF | Canon, Japan |
| Laboratory tests | Hematology | XE-2100 and XE-5000 | Sysmex, Japan |
|  |  | Alifax Test 1 | ALIFAX, Italy |
|  | Urine Test | UF5000 | Sysmex, Japan |
|  |  | UC3500 | Sysmex, Japan |
|  | Stool Test | FA160 | Orienter, China |
|  | Liver Function Tests | cobas C702 | Roche, Switzerland |
|  | Kidney Function Test | cobas C702 | Roche, Switzerland |
|  | Glucose Metabolism | cobas C702 | Roche, Switzerland |
|  |  | HLC-723G8 | TOSOH, China |
|  |  | ADAMS A1C HA-8190V | аrKгау, Japan |
|  |  | capillarys 3 | sebia, France |
|  | Tumor Markers | cobas 8000 e801 | Roche, Switzerland |
|  | Lipid Profile | cobas C702 | Roche, Switzerland |
|  | Inflammation and Immunology | IMMAGE 800 | BECKMAN COULTER, USA |
|  |  | FACSCantoⅡ | Becton Dickinson, USA |
|  |  | FACSCantoplus | Becton Dickinson, USA |
|  | Infectious Diseases | SLAN-96 Real-time PCR Instrument | HONGSHI, China |
|  |  | Luminex-200 Multiplex Flow Cytometer | Luminex, USA |
|  |  | CL-8000i | Mindray, China |
|  |  | cobas e 801 | Roche, Switzerland |
|  | Hormones | cobas C702 | Roche, Switzerland |
|  |  | cobas 8000 e 801 | Roche, Switzerland |
|  | Gastrointestinal System | URANUS AE 115 | AIKANG, China |
|  | Electrolytes and Minerals | cobas C702 | Roche, Switzerland |
|  | Thyroid function Tests | cobas 8000 e 801 | Roche, Switzerland |
|  | Other Biochemical Tests | cobas 8000 c702 | Roche, Switzerland |
|  |  | cobas e 602 | Roche, Switzerland |
|  |  | cobas 8000 e801 | Roche, Switzerland |
| Imaging tests | Supersonic inspection | EPIQ5 | PHILLIPS, USA |
|  | Computed Tomography | SOMATOM Definition AS 128 | Siemens, Germany |
|  |  | uCT960+ | United-Imaging, China |
|  |  | Somatom Definition Flash | Siemens, Germany |
|  |  | Revolution CT ES | GE, USA |
|  | X-ray examination | uDR780i | United-Imaging, China |
|  | Electrocardiogram | iMAC120 | ZONCARE, China |
|  |  | SE-1026 | EDAN, China |

## Supplementary Table 5. Measurement tools in questionnaires

| Interested factors or aspects | Measurement tool | |
| --- | --- | --- |
|  | In the WHALE Database | In the WHALE Health Trajectory Cohort (prospectively) |
| **Demographics** | **8 questions:** age, gender, nation, marriage status, education level, family income, residence, occupation | |
| **Lifestyle** | | |
| Smoking | **5 questions:** current cigarette smoking status, daily and annual cigarette consumption, frequency of passive smoking, and exposure to kitchen fumes | **8 questions:** current cigarette smoking status, the age at smoking starting both for current smokers and past smokers, daily cigarette consumption both for current smokers and past smokers, passive smoking status, frequency and duration of passive smoking |
| Alcohol drinking | **3 questions:** current drinking status, the average amount of alcohol consumed per occasion, and the duration of drinking history | **4 questions:** current drinking status, frequency of alcohol drinking, the average amount of alcohol consumed per day, the types of alcohol consumed |
| Diet | **7 questions:** the extent of salt, oil, and spicy ingredients used in their diet, the primary types of meat consumed and their quantities, the frequency of smoked foods consumption, the frequency of pickles consumption, the frequency of tea consumption | **7 questions:** meat and vegetable balance in the diet, dietary preferences regarding the taste profile, frequency of spicy food consumption in the past month, preference for spiciness level, ingredients for spiciness, frequency of numbing food that contain Sichuan pepper consumption, ingredients for numbness |
| Physical activity and sedentary bahevior | **6 questions:** working hours, sedentary time, exercise frequency, duration of physical activity, the intensity levels of work and exercise | **International Physical Activity Questionnaire (IPAQ) short form (16 items):** four dimensions: vigorous physical activity, moderate physical activity, walking, and sedentary time |
| **Psychological conditions** | | |
| Sleep quality | **1 question:** subjective sleep quality  **Pittsburgh Sleep Quality Index (PSQI) (19 items):** further evaluation on subjective sleep quality, sleep latency, sleep duration, habitual sleep efficiency, sleep disturbances, use of sleep mediations, and daytime dysfunction during the past week | |
| Anxiety | **The Self-Rating Anxiety Scale (SAS) (20 items)** | **the Patient Health Questionnaire-9 (PHQ-9) (9 items)** |
| Depression | **the Self-Rating Depression Scale (SDS) (20 items)** | **the Generalized Anxiety Disorder Scale (GAD-7) (7 items)** |
| **Health assessment** | | |
| Medical and family history of chronic diseases | including hypertension, diabetes, coronary heart disease, stroke, gout, rheumatoid arthritis, pulmonary tuberculosis, chronic obstructive pulmonary disease, cancer, chronic hepatitis, asthma, gastritis, gallstones, non-traumatic fracture, hyperthyroidism, hypothyroidism, thyroid Nodules | |
| Medication use history | covering 3 types of medication | duration, starting year, adherence of medication use covering 6 categories |

## Supplementary Figure 2. The volume range of plasma in each sample tube


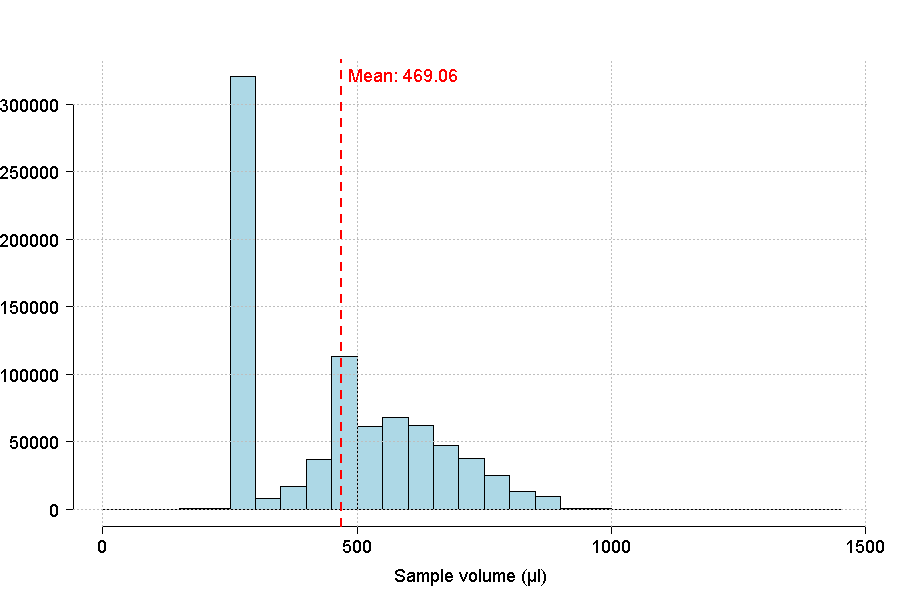


## Supplementary Figure 3. Flowchart of data clean


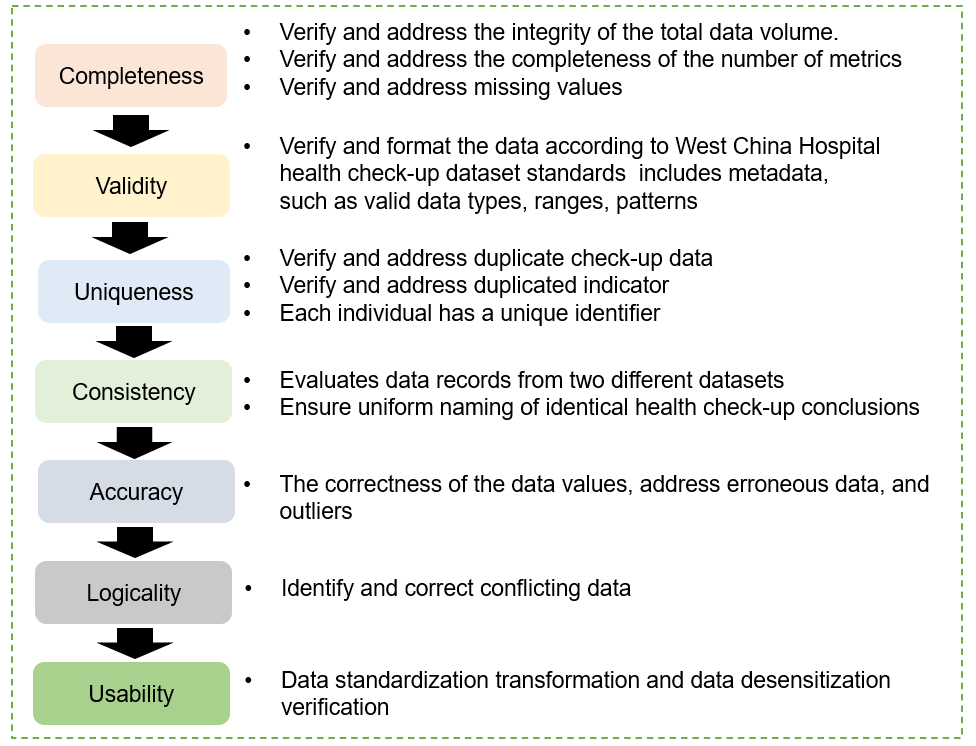


## Supplementary Table 6. Comparative summary table contrasting WHALE with other major cohorts

| Cohort | Geographic | Ethnic Diversity | Sample Size | Types of Data Collected | Follow-up Design and Frequency | Distinctive features of WHALE Study |
| --- | --- | --- | --- | --- | --- | --- |
| WHALE Study | Sichuan Province, China (strategically positioned between eastern and western China, one of China’s most populous provinces) | 85.6% Han, 14.4% others | 1,526,686 (WHALE Database,); 273,628 (Health Trajectory Cohort,) | Health checkup data (body measurement, sociodemographic, laboratory tests, imaging); EHR data, questionnaires (lifestyle, health status), biological samples, genomic data | Ambispective cohort; biennial active follow-up; passive via healthcare/admin database linkage | NA |
| Our Future Health | UK-wide (England, Scotland, Wales; Northern Ireland from 2025) | Diverse UK population, ethnicity not specified | 2 million consented (1.4 million questionnaires, 1.1 million blood samples) | Questionnaires (demographics, lifestyle), physical measures, blood (plasma, DNA), genomic data, linked health records | Continuous passive follow-up via health record linkage; active re-contact studies planned for 2025 | WHALE Study 's active biennial follow-ups and multimodal data collection (biospecimens, imaging, clinical records) provide deeper insights into health trajectories. |
| All of Us | US-wide, >340 sites | 51% non-white, 80% underrepresented groups | 230,000 total, 175,000 core (biosamples, measures, EHR) by Jul 2019 | EHRs (billing, labs, meds), questionnaires, physical measures, biosamples (blood/saliva), genomic data, Fitbit | Passive follow-up via EHR and linked data (death index, pharmacy, claims); optional surveys and biospecimen collections, no fixed schedule | WHALE Study offers structured biennial active follow-ups, standardized extensive imaging data, and detailed physical examination records. |
| MVP | US-wide, ~50 VA medical facilities | 77.2% White, 13.5% African American, 7.6% Other, 1.6% not reported | 397,104 enrolled by Aug 2015 | Questionnaires (baseline, lifestyle), VA EHRs, blood for genetic/other assays | Passive follow-up via VA EHRs for health events, mortality; active via surveys or re-contact as needed | WHALE Study includes diverse Chinese ethnic populations, routine collection of comprehensive physical and laboratory data, and structured biennial follow-ups. |
| UK Biobank | UK-wide, 22 assessment centres | 94.2% White, 1.9% Asian, 1.6% Black, 2.3% Other/Unknown | 500,000 (2006-2010). | Questionnaires (sociodemographic, lifestyle, health), physical measures, biosamples (blood, urine, saliva), accelerometry, multimodal imaging, genome-wide genotyping | Passive follow-up via health record linkage; active via repeat imaging, seasonal monitoring for some. No fixed schedule. | WHALE Study emphasizes repeated longitudinal health check-ups (up to >10 visits for ~3% participants) integrated within a hospital network, providing richer temporal data. |
| China Kadoorie Biobank (CKB) | 10 regions (5 urban, 5 rural) across China | Primarily Han Chinese, specific ethnic diversity not detailed | ~512,891 (2004-2008) | Questionnaires (sociodemographic, lifestyle, medical history), physical measurements, blood samples (plasma, DNA), linked health records | Continuous passive follow-up via linkage with mortality/morbidity registries and national health insurance databases; active re-survey of ~25,000 participants | WHALE Study incorporates active follow-ups combined with passive linkage, extensive imaging and repeated clinical/laboratory assessments across hospital settings(alliance network). |
| Kailuan Study | Tangshan, Hebei Province | Primarily Han Chinese, specific ethnic diversity not detailed | ~100,000 (2006-2007) | Physical exams, laboratory tests, questionnaires (lifestyle, occupational health), limited biospecimens | Biennial health check-ups; passive linkage to health records | WHALE Study offers greater sample diversity, broader disease scope, more comprehensive biological sample collections, and longitudinal imaging data. |
| Taizhou Longitudinal Study | Taizhou, Jiangsu, China (3 districts) | Primarily Han Chinese, specific ethnic diversity not detailed | ~200,000 (baseline 2007) | Questionnaires (socioeconomic, lifestyle, diet, medical history), physical measurements, biological samples (buccal cells, fasting blood) | Active follow-up every 3 years with nested case-control approach; passive monitoring via chronic disease registry | WHALE Study's biennial active follow-up, extensive multimodal data, and frequent repeated measurements provide more detailed longitudinal health trajectories. |

EHR = Electronic Health Records; MVP = Million Veteran Program; VA = Veterans Affairs; CKB = China Kadoorie Biobank.
